# The Endocannabinoids-Microbiota Partnership in Gut-Brain Axis Homeostasis: Implications for Autism Spectrum Disorders

**DOI:** 10.3389/fphar.2022.869606

**Published:** 2022-06-03

**Authors:** Roberto Coccurello, Maria Cristina Marrone, Mauro Maccarrone

**Affiliations:** ^1^ Institute for Complex Systems (ISC), National Council of Research (CNR), Rome, Italy; ^2^ European Center for Brain Research/Santa Lucia Foundation IRCCS, Rome, Italy; ^3^ Ministry of University and Research, Mission Unity for Recovery and Resilience Plan, Rome, Italy; ^4^ Department of Biotechnological and Applied Clinical and Sciences, University of L’Aquila, L’Aquila, Italy

**Keywords:** gut-brain axis, microbiota, endocannabinoids, N-acyl-ethanolamines, autism spectrum disorders, microglia, immune system

## Abstract

The latest years have witnessed a growing interest towards the relationship between neuropsychiatric disease in children with autism spectrum disorders (ASD) and severe alterations in gut microbiota composition. In parallel, an increasing literature has focused the attention towards the association between derangement of the endocannabinoids machinery and some mechanisms and symptoms identified in ASD pathophysiology, such as alteration of neural development, immune system dysfunction, defective social interaction and stereotypic behavior. In this narrative review, we put together the vast ground of endocannabinoids and their partnership with gut microbiota, pursuing the hypothesis that the crosstalk between these two complex homeostatic systems (bioactive lipid mediators, receptors, biosynthetic and hydrolytic enzymes and the entire bacterial gut ecosystem, signaling molecules, metabolites and short chain fatty acids) may disclose new ideas and functional connections for the development of synergic treatments combining “gut-therapy,” nutritional intervention and pharmacological approaches. The two separate domains of the literature have been examined looking for all the plausible (and so far known) overlapping points, describing the mutual changes induced by acting either on the endocannabinoid system or on gut bacteria population and their relevance for the understanding of ASD pathophysiology. Both human pathology and symptoms relief in ASD subjects, as well as multiple ASD-like animal models, have been taken into consideration in order to provide evidence of the relevance of the endocannabinoids-microbiota crosstalk in this major neurodevelopmental disorder.

## 1 Endocannabinoids and the Gut Microbial Ecosystem

### 1.1 Endocannabinoids: A Brief Dip Into the Lipid Pot

In essence, endocannabinoids (eCBs) are bioactive lipid mediators that are produced from the common precursor arachidonic acid (ARA), and therefore belong to the family of polyunsaturated *n*-6 fatty acids (*n*-6 PUFAs) (Cascio, 2013; Bisogno and Maccarrone, 2014). eCBs exert pleiotropic actions and are able to regulate multiple biological functions, as demonstrated in neuropsychiatric disorders (NPDs) (Navarrete et al., 2020) and in the regulation of body weight and feeding (Horn et al., 2018), by the way of their action as high-affinity ligands for specific G protein-coupled receptors (GPCRs) (Pertwee, 2015). *N*-arachidonoyl-ethanolamine [anandamide (AEA)] and 2-arachidonoylglycerol (2-AG) are well-studied eCBs that bind with high affinity type 1 and type 2 cannabinoids receptors (CB_1_ and CB_2_, respectively), two GPCRs. Together, the endogenous ligands (i.e., AEA and 2-AG), CB_1_/CB_2_ receptors and the enzymes accountable for eCBs synthesis [N-acyl-phosphatidylethanolamine-specific phospholipase D (NAPE-PLD) and diacylglycerol lipase (DAGL)-α and -β, for AEA and 2-AG respectively], degradation [fatty acid amide hydrolase (FAAH) and monoacylglycerol lipase (MAGL), for AEA and 2-AG respectively], transport and, ultimately, eCBs concentration and tissue availability, give rise to the so-called eCB system (Mechoulam and Parker, 2013; Pertwee, 2015).

In turn, AEA and 2-AG are part of two larger lipid groups of eCBs-like molecules including *N*-acyl-ethanolamines (NAEs) and 2-acylglycerols (2-AcGs), respectively (Bisogno and Maccarrone, 2014; Di Marzo, 2018). NAEs and 2-AcGs share the common biosynthetic pathways involved in either AEA or 2-AG formation or in their inactivation, but none of their members do electively activate CB_1_/CB_2_ receptors (Di Marzo et al., 1994; Bisogno et al., 2003; Bisogno and Maccarrone, 2014). In the NAEs family there are several bioactive lipids such as the *N*-oleoylethanolamine (OEA, C18:1) and the *N*-palmitoylethanolamine (PEA, C16:0), which are produced from oleic acid (OA) or palmitic acid (PA), respectively (Schmid, 2000), as well as the *N*-linoleoylethanolamine (LEA, C18:2) and the *N*-stearoylethanolamide (SEA, C18:0) (Ezzili et al., 2010). On the other hand, in the 2-AcGs family are included lipid congeners with fatty acids of different length such as 2-oleoyl-glycerol (2-OG), 2-linoleoyl-glycerol (2-LG) and 2-palmitoylglycerol (2-PG) (Murataeva et al., 2016). More abundant in most animal tissues than AEA (Tsuboi et al., 2018), the other NAEs show different physiological actions by targeting multiple non-eCB receptors. PEA is a lipid signaling molecule exerting a well described anti-inflammatory, analgesic and neuroprotective activities (Lambert et al., 2012; Mattace Raso et al., 2014), whose effects are in part mediated by the activation of the nuclear peroxisome proliferator-activated receptor-α (PPAR-α) (LoVerme et al., 2005) as well as by the potentiation of AEA action (i.e., the “entourage effect”) at the transient receptor potential vanilloid type-1 (TRPV1) channels (De Petrocellis et al., 2001), or else *via* the contribution of PPAR-γ and PPAR-δ isoforms (Costa et al., 2008; Paterniti et al., 2013). OEA is an anorexigenic, neuroprotective and lipolytic satiety factor (Rodríguez de Fonseca et al., 2001; Guzmán et al., 2004; Provensi et al., 2014) that achieves these effects *via* the activation of PPAR-α receptors. Although with a lesser affinity than PEA, also OEA has been reported to possess agonistic activity at the TRPV1 channels, thus increasing TRPV1-evoked currents (Ahern, 2003; Wang et al., 2005). Moreover, both PEA and OEA, as well as LEA, are able to bind the lipid-activated receptor of the enteroendocrine cells GPR 119 (Overton et al., 2006; Hansen et al., 2011). This ability may be of key importance for the GPR119-mediated intestinal secretion of the incretin hormone glucagon-like peptide-1 (GLP-1), and therefore for the maintenance of insulin secretion/sensitivity and appetite suppression not only in metabolic pathology such as diabetes and obesity but also in neurodegenerative disorders (Brown et al., 2018; Müller et al., 2019).

### 1.2 Gut Microbial Composition and eCB Signaling: The Powerful Role of Dietary Fat Composition

It is widely recognized that the large gut microbial population, generally named microbiota, is composed of about 100 trillions of microorganisms mainly including anaerobic bacteria, fungi and protozoa (Ley et al., 2006; Matijašić et al., 2020). Firmicutes, Bacteroidetes, Proteobacteria and Actinobacteria are the most copious and representative bacterial species, living in symbiotic “harmony” with the host and significantly contributing to its metabolic and immune functions as well as to the preservation of integrity of the intestinal epithelium (Burcelin, 2016). Such diversity and sensitive symbiotic ecosystem can be selectively perturbed by different lifestyles and nutritional challenges. The two main abundant phyla Firmicutes and Bacteroidetes are also the most susceptible to the shaping action of low- vs. high-fat diet, high-sugar diet and ingestion of different fatty acids, as showed by the incidence of dysbiosis and the higher representation of Firmicutes phyla (e.g., Lactobacillaceae, Veillonellaceae, and Lachnospiraceae) in obesity (Crovesy et al., 2020).

**FIGURE 1:**
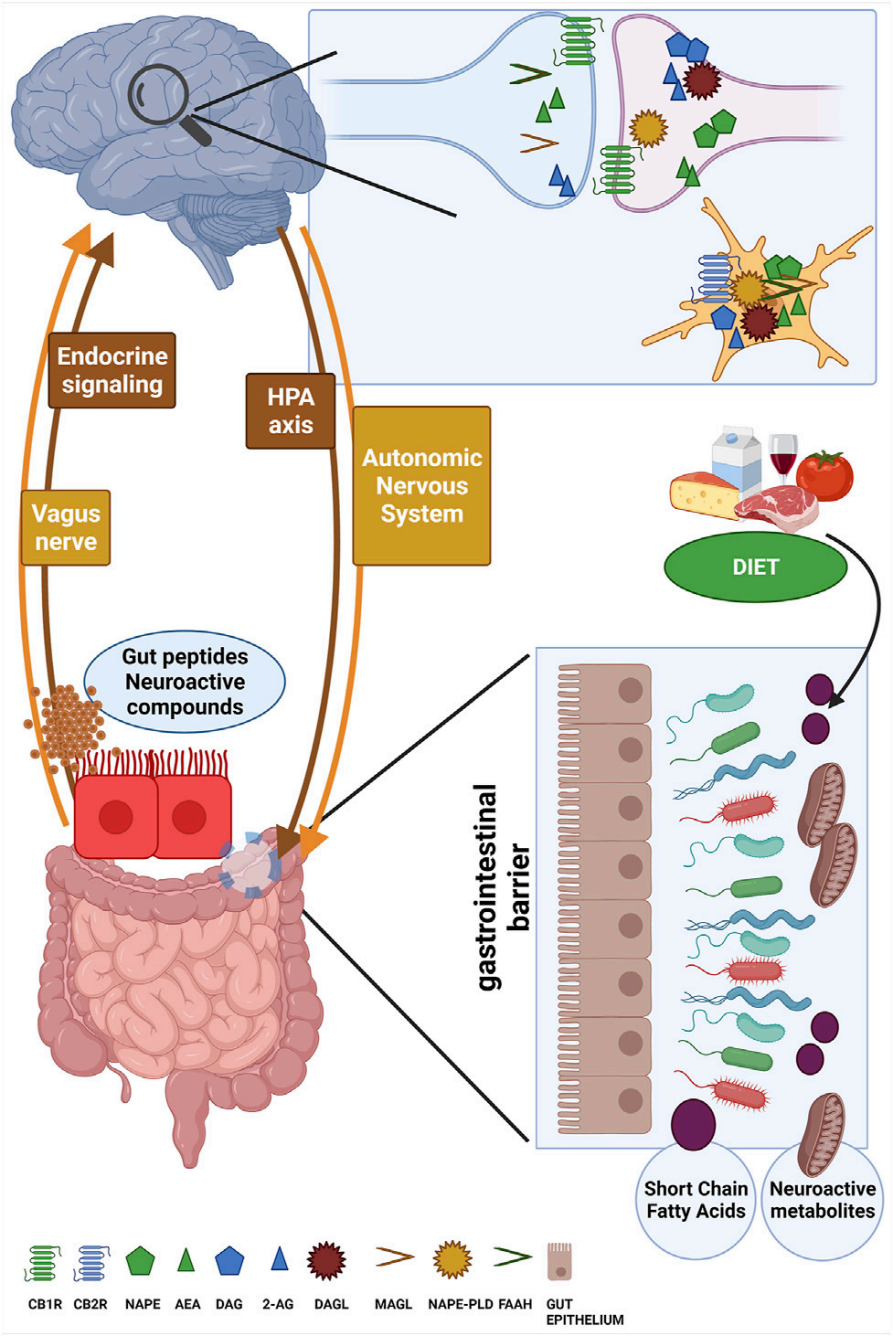
The figure schematizes the bi-directional and reciprocal influence between brain and gut (i.e., the gut-brain axis) along the main routes of communication such as the HPA axis, the autonomic nervous system, the vagus nerve and endocrine signaling. The ability to sense microbiota metabolites, gut peptides and neuroactive compounds and to transfer to the brain this complex information about the ongoing status of gut microbial ecosystem is fundamental for the generation of adaptive or maladaptive responses, for instance by the HPA axis and cortisol secretion. Here, the main components of the eCB system machinery are depicted at both presynaptic and postsynaptic terminals as well as in microglia cells, and the attention is focused on the role of powerful external modifiers such as dietary composition to mutually affect gut microbiota composition and eCB signaling. created in BioRender.com.

Since eCBs and NAEs are bioactive lipids, their cell and tissue availability can be directly changed by dietary habits and, in particular, by dietary fatty acids composition (Chianese et al., 2017). Indeed, while the *n*-6 carbon essential fatty acid linoleic acid (LA, 18:2, *n*-6) is the precursor for ARA biosynthesis (and then for AEA and 2-AG production), the *n*-3 carbon essential fatty acid α-linolenic acid (ALA, 18:3, *n*-3) is the precursor of the main *n*-3 PUFAs docosahexaenoic acid (DHA) and eicosapentaenoic acid (EPA) from which active metabolites such as *N*-docosahexaenoyl-ethanolamine (DHEA) and *N*-eicosapentaenoyl-ethanolamine (EPEA) are also produced (Berger et al., 2001; Maccarrone et al., 2010a). It is well-established that the *n*-6/*n*-3 ratio has been dramatically altered by the worldwide adoption of the Western diet (WD) lifestyle especially in the last 50 years, shifting from about 5:1 to the currently estimated 20:1 ratio (Simopoulos, 2006; Blasbalg et al., 2011). A general scheme of the gut-brain interface, including the major pathways of communication involved, the key components of the eCB system and the influence of dietary factors is showed in Figure 1.

Increasing the intake of LA might alter the eCBs levels (Berger et al., 2001; Alvheim et al., 2013; Alvheim et al., 2014), and CB_1_ receptor function in selected brain areas (prefrontal cortex and nucleus accumbens) can be permanently altered by feeding animals with a *n*-3 deficient diet during gestation and lactation (Lafourcade et al., 2011). Moreover, there is evidence that the beneficial impact on cognition, or the attenuation of cognitive decline provided by the decrease of dietary *n*-6/*n*-3 ratio *via n*-3 PUFAs dietary supplementation can be preserved both during adolescence and adulthood (Muldoon et al., 2010; Luchtman and Song, 2013), although negative results have also been reported (Stough et al., 2012). The anti-neuroinflammatory and anti-depressive potential of DHA and EPA has been studied in the recent literature in both animal models (Minogue et al., 2007; Moranis et al., 2012) and clinical reports (Rees et al., 2006; Liao et al., 2019), along with their use in dietary supplementation as nutritional guideline to slow down age-associated cognitive decline through preservation of neurogenesis and synaptic plasticity (Dyall, 2015). The levels of dietary LA can affect brain content of 2-AG and AEA and increase liability to obesity (Massiera et al., 2010; Alvheim et al., 2012) but, on the other hand, the different composition of dietary fatty acids can be exploited as anti-obesity agent by both reduction of 2-AG levels (Banni et al., 2011) and the modulation of NAEs concentrations (Pu et al., 2016). Most importantly, the composition of dietary *n*-3 and *n*-6 PUFAs and therefore ALA or LA is able to shape the huge microbial community composing the gut microbiota (Marrone and Coccurello, 2019; Wolters et al., 2019). Gut dysbiosis, low-grade inflammation and gut permeability are indeed leading causes for obesity development (Levy et al., 2017; Sun et al., 2018). In obesity, the systemic elevation of the eCB tone (brain included) as well as the upregulation of the CB_1_ receptor in adipose tissue, liver and muscle have been extensively demonstrated (Matias and Di Marzo, 2006; Starowicz et al., 2008; Silvestri and Di Marzo, 2013). Accordingly, blockade of CB_1_ receptors may revert both systemic inflammation and dysbiosis induced in obese mice by chronic high-fat diet (HFD) regimen (Mehrpouya-Bahrami et al., 2017).

In support of the idea of a mutual interconnection between endocannabinoids and microbiota, especially in the regulation of energy metabolism (Cani et al., 2016), it has been pointed out that human microbiota can encode *N*-acyl amides (i.e., eCBs congeners) that can bind G-protein-coupled receptors (GPCRs), thus modulating the intestinal function by the way of the GPR119 (Cohen et al., 2017). Dietary habits and composition are the most important modifiers of the intestinal microbial community (Rinninella et al., 2019), and the eCB machinery is high susceptible to changes in food composition and dietary patterns (Bisogno and Maccarrone, 2014; Passani and Coccurello, 2016; Chianese et al., 2017). In turn, food-induced changes in the eCB system can be paralleled by changes in gut microbiota composition. Switching for only 3 days to a high-fat/high-sucrose diet can produce in the small intestine an increase of AEA and 2-AG together with changes in the abundance of selected bacteria that were found to correlate to blood levels of eCB mediators such as AEA and DHEA (Lacroix et al., 2019). Both eCBs and NAEs plasma levels are directly modulated by nutrients and diet composition, as demonstrated by the switch to a Mediterranean dietary regimen in obese or overweight subjects (Tagliamonte et al., 2021) that reduced AEA levels while increased OEA/PEA and OEA/AEA ratios. Interestingly, the shift from Western-to-Mediterranean diet also produced an increase of faecal content of the mucin-degrading Gram-negative bacterium of the phylum Verrucomicrobia *Akkermansia muciniphila* (*A. muciniphila*), whose abundance appears inversely associated with gut dysbiosis and gut permeability (Lopetuso et al., 2020) and therefore is associated to healthy microbial ecology. As “gatekeeper” of intestinal mucosa and gut health, the abundance of *A. muciniphila* can be modulated by diet and nutrients as demonstrated by fructo-oligosaccharides prebiotic supplementation (Everard et al., 2013). However, not only the fluctuations in *A. muciniphila* gut abundance are paradigmatic of the tight relationship between diet, gut microbial ecology and (as afterwards discussed) eCB-like lipid mediators (Depommier et al., 2021), but also of the alterations found in gut microbiota of subjects with autism spectrum disorder (ASD). The direct, as well as indirect, impact of macronutrients (mostly fats) on the eCB system of the gastrointestinal (GI) tract and gut-brain signaling have been recently and comprehensively analyzed, especially for the implication in metabolic disease (Di Patrizio, 2021). In metabolic disease, but also in other clinical conditions, there is evidence that the alteration of selected microbial populations and the administration of eCB or eCB-like lipids may help to rebalance gut bacterial composition. As for instance, vitamin D deficiency was shown to exacerbate neuropathic pain, reducing both gut microbial diversity and 2-AG colon levels, while the supplementation of vitamin D and PEA was shown to attenuate allodynia and rebalance the Firmicutes/Bacteroidetes ratio, including the increase of gut *Akkermansia* species (Guida et al., 2020).

In this context, a novel route for a future investigation of the relationship between ASD, microbiota and eCB system might also be identified in the analysis of the eating problems/disorders reported in ASD subjects. Indeed, unhealthy eating habits are observed in subjects with ASD in which the incidence of eating disorders (EDs) is much greater than in the general population (Sharp et al., 2013). The higher incidence of EDs and alterations of eating behavior and food selection in ASD patients (Råstam et al., 2013; Sharp et al., 2013; Nickel et al., 2019), suggest to investigate whether in addition to cognitive endophenotypes (Zucker et al., 2007) there might be common gut microbial signatures in EDs and autism along with parallel changes in eCB signaling. A surprising variety of different eating-associated abnormalities afflict children and adolescents who have received a diagnosis of autism. The eating and feeding disorders described span from the more “canonical” EDs (i.e., AN, BN, and BED) to rumination disorder, picky eating, avoidant/restrictive food disorder (ARFID) as well as Pica (i.e., insistent eating of non-food substances) (American Psychiatric Association Diagnostic and Statistical Manual of Mental Disorders (DSM-IV), 2012; American Psychiatric Association, 2013; Fields et al., 2021; Inoue et al., 2021). For this reason, these aspects are suggested but not further developed in the present work, hoping that other studies will address in the next future the fascinating issue of multiple dietary approach in ASD (e.g., gluten-free diet or ketogenic diet) and parallel impact on eCB system/gut microbiota interplay.

## 2 eCBs-Microbiota Reciprocal Contamination: Implications for Autism Spectrum Disorder Pathophysiology

Up to now, a limited, but significant number of studies, has provided evidence that mood disorders can be associated with marked changes of eCB signaling that can be mechanistically linked to depressive-like behaviors and alterations of microbiota environment. Although the relationship between gut dysbiosis and NPDs is increasingly documented (Fond et al., 2015; Cenit et al., 2017; Marrone and Coccurello, 2019), evidence for the involvement of eCB-microbiota axis in mood and neurodevelopmental disorders (NDDs) are much more limited. By contrast, recent studies have add evidence in support of the idea that alteration of the eCB signaling system can explain that is possible to use fecal microbiota transplantation (FMT) to transfer depression-like symptoms from “donors” to recipient mice (Chevalier et al., 2020). In particular, this study shows that mice receiving FMT from chronically-stressed mice develop similar depression-like symptoms and concomitant deficits in AA availability, 2-AG synthesis, hippocampal eCB signaling and adult neurogenesis, and that all these effects can be counteracted by supplementation of probiotic *Lactobacillus* (i.e., *L. plantarum Lp* ^*WJL*^) (Chevalier et al., 2020). In agreement, there is also the elegant demonstration that antibiotic-induced derangement of microbiota composition and alterations of specific members of the eCB system are accompanied by depressive-like behaviors including deficits of social behavior, neuroinflammation and changes of neuronal-glia-microglia communication in the hippocampus (Guida et al., 2018). This study shows that antibiotic exposure increases Proteobacteria and Actinobacteria, while probiotic treatment with *Lactobacillus casei* DG increases *Lachnospiraceae* and reduces *Enterococcaceae* and *Bacillaceae*, thus mitigating the morphological alterations observed in hippocampal astrocytes and microglia. Notably, antibiotic-induced dysbiosis was associated with a marked reduction in the small intestine of two eCBs members, one of them (i.e., *N*-arachidonoylserotonin (AA-5-HT) involved in the inhibition of FAAH and the TRPV1 channel as well as in antidepressant activity (Navarria et al., 2014), which resulted normalized by probiotic treatment (Guida et al., 2018). The major alterations of eCBs and eCB-like lipid mediators in the brain widely reported in germ free (GF) mice (Manca et al., 2020b), further corroborate the idea that the eCB signaling system mediates many functions of the bidirectional microbiota-gut-brain communication. Even beyond the implications for NDDs, there is now evidence that the complex homeostatic role exerted by the eCB system and eCB-like mediators is including unpredicted functions such as those recently described for the intestinal expression of the liver-expressed antimicrobial peptide 2 (LEAP2), which is partially regulated by eCB system-microbiota interaction and is important for the impact of nutrients on energy metabolism (Shen et al., 2022).

**FIGURE 2:**
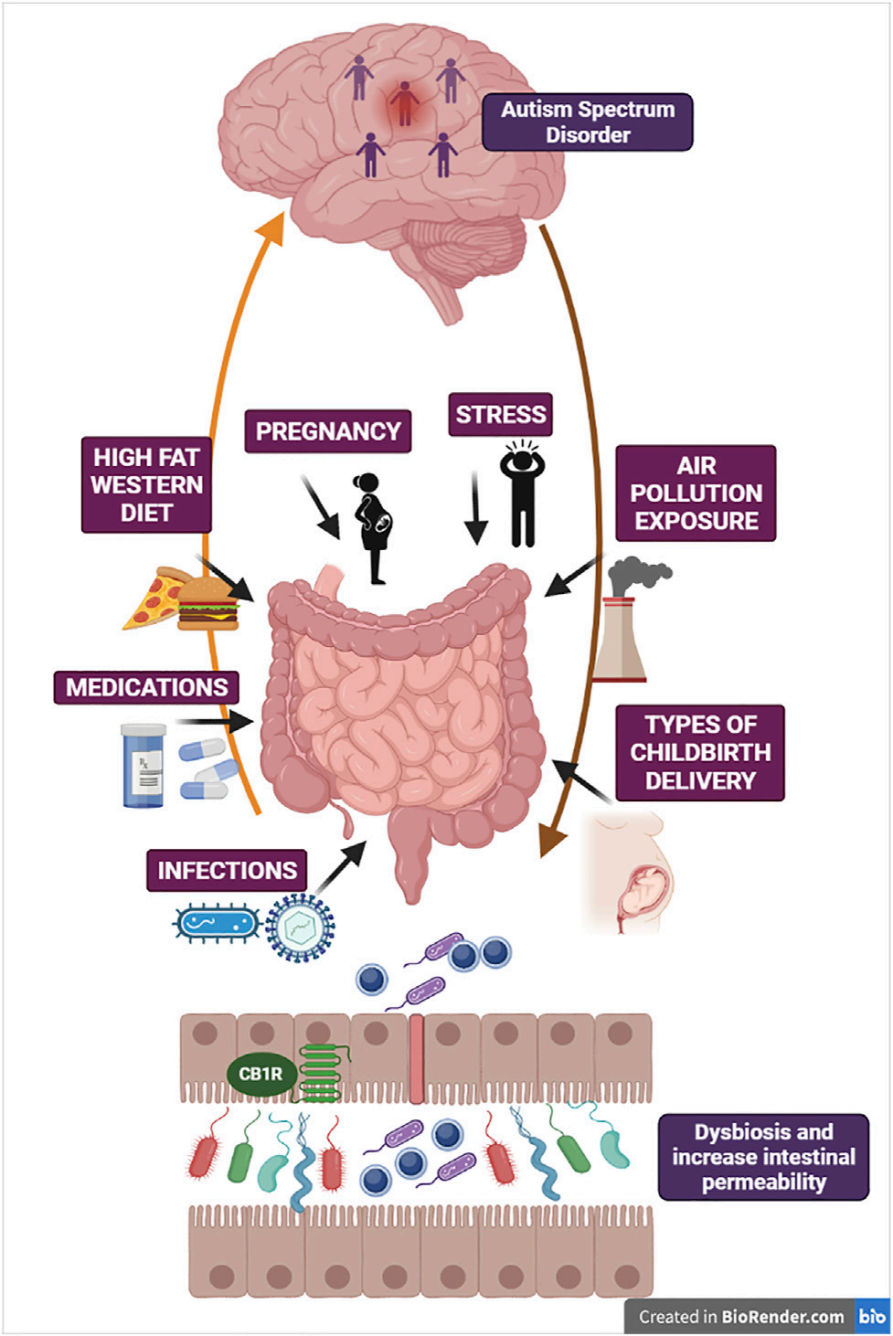
The figure illustrates some critical mechanisms involved in derangement of gut microbiota ecosystem as potential threats increasing susceptibility to ASD. Gut microbiota and brain are reciprocally interconnected *via* multiple neural, endocrine and immune bidirectional pathways. Here are schematized brain-gut efferent pathways (e.g., HPA axis, autonomic nervous system) and gut-to-brain afferent pathways (e.g., vagal innervation and enteroendocrine signaling). Changes in gut microbiota composition and dysbiosis can be attributed to multiple threats, such as deleterious obesogenic diets (Western diet/high-fat diet), psychosocial and physical stressors, chronic medications due to medical co-morbidities, infections of multiple origins and exposure to air pollution. These environmental challenges may be viewed as pathogenetic factors not only after birth, during postnatal neurodevelopment, but also during maternal gestation with relevance for infections occurring during pregnancy and the different types of childbirth delivery. Under the pressure of multiple threats, abnormal gut function and dysbiosis with pathobiont overgrowth may became the prevalent inflammatory condition, thus altering brain-gut axis reciprocal signaling and brain response. In turn, progressive loss of intestinal barrier function and consequent *leaky gut* increases the transfer of detrimental bacterial components to systemic circulation, intensifying chronic inflammation and susceptibility to ASD. The figure also depicts CB_1_ receptors located at the intestinal epithelium, in proximity with neuroendocrine cells, which imply the role of eCB machinery in the regulation of gut barrier integrity.

ASD is one of the most challenging, disabling and heterogeneous NDD, characterized by the impairment in social communication and social interaction, limited interests, repetitive and stereotyped patterns of behaviors (American Psychiatric Association Diagnostic and Statistical Manual of Mental Disorders (DSM-IV), 2012). The significant association between abnormal changes in gut microbiota, GI disorders and ASD symptoms provides the strong rationale to consider the severe alteration of the microbial gut community of ASD subjects as an important pathogenetic factor in ASD development. The number of evidence supporting the concept of gut dysbiosis in ASD is constantly growing, as for instance by the demonstration that GM mice receiving microbiota samples from ASD human donors develop ASD-like symptoms (Sharon et al., 2019). Before introducing the impact of the eCBs complexity in the brain-gut microbiota axis/ASD pathophysiology, we suggest to refer to Figure 2 that provides a schematic idea of the different environmental challenges and multiple threats responsible of gut dysbiosis and increasing risk of ASD during the neurodevelopment. Under prenatal and postnatal burden, abnormal gut function and dysbiosis may became a prevalent inflammatory condition leading to an alteration of brain-gut axis signaling. As consequence, the loss of intestinal integrity and leaky gut causes detrimental bacterial components transfer to circulation, increasing risk and susceptibility to ASD. The eCB system establishes a tight functional interaction with the gut microbiota ecosystem and participate to the regulation of neuroepithelial gut barrier physiology contributing to determine intestinal permeability *via* CB_1_ receptor activation within the intestinal epithelium and/or enhancement of AEA/2-AG signaling (Karwad et al., 2017). Accordingly, blockade of CB_1_ receptors attenuates inflammation in gut microbiota and diet-induced obesity intestinal permeability (Mehrpouya-Bahrami et al., 2017). Indeed, GM mice show severe alterations of intestinal eCBs gene expression and consequent changes of eCB signaling (Manca et al., 2020a).

Thus, the eCBs-microbiota partnership could have a distinctive role in the ASD, both for the understanding of its complex pathogenesis as well as in relation to the discovery of novel therapeutics. In this context, the mutual communication between the microbiota ecosystem and eCB signaling appears represented by the ASD, in which microbiota dysfunction and changes in the eCB system have been described either in association or as independent “causal” factors. The so-called “recreational” use of *Cannabis sativa* and, consequently, the empirical experience that its consumption in a variety of different forms (e.g., fiber hemp, seeds and female flowers) and modalities (e.g., ingestion, inhalation and smoking) dramatically affect human social behavior is known from thousands of years (Ren et al., 2019). Although social behavior is generally self-assessed by *Cannabis* users, the most part of subjective feelings reported converge towards the description of pro-social behaviors enhancement, including more social interactions, less agonistic actions and hostility, a greater involvement in social play, higher sociability such as empathy and altruism, and less social anxiety (Wei et al., 2017). Nevertheless, *Cannabis* chronic consumption/intake is also associated with important psychoactive effects provoking major concerns in clinicians, especially at the light of the significant (though not yet prevalent) and growing consensus around the medical use of *Cannabis* (Hall et al., 2019). As recognized, the adverse effects on mood, anxiety and cognition are almost exclusively imputable to the main psychoactive component of the *Cannabis* Δ-9-tetrahydrocannabinol (THC) (Dow-Edwards and Silva, 2017), which makes impossible the mere hypothesis of *Cannabis’s* use for medical purpose, and particularly in the case of developing brains, as for all NDDs. Among the over hundred phytocannabinoids present in the *Cannabis* plant, cannabidiol (CBD) has attracted a great interest for the lack of psychoactive effects (Izzo et al., 2009) showing even the ability to antagonize some psychotropic effects of THC (Niesink and van Laar, 2013). Indeed, CBD is a non-cannabimimetic component that does not bind (i.e., has a very low affinity) CB_1_ and CB_2_ receptors, while it exerts inverse agonism at CB_2_ receptors (Thomas et al., 2007) and negative allosteric modulatory activity at CB_1_ receptors (Laprairie et al., 2015). Additional pharmacological features describe the CBD as a chemical compound able to inhibit, *via* FAAH, AEA hydrolysis and produce antipsychotic effects (Leweke et al., 2012), and a phytocannabinoid with agonistic activity at 5-HT1A receptor (Russo et al., 2005) and desensitization activity of TRPV1 (Bisogno et al., 2001), thus providing anti-inflammatory, analgesic, anti-depressant and anxiolytic effects (Schier et al., 2014; Shannon et al., 2019; Atalay et al., 2020). Interestingly, CBD also binds the orphan G protein-coupled receptor 55 (GPR55), a recently included new member of the eCB family (Sharir and Abood, 2010), showing an involvement in several mechanisms accountable for the exacerbation of inflammatory processes (Yang et al., 2016). GPR55 is densely distributed all along the GI tract where its expression increase in case of intestinal inflammation, which can be relieved by its pharmacological blockade (Stančić et al., 2015). Of note, a partial antagonistic activity has been described for CBD at the GPR55 (Ryberg et al., 2007), thus disclosing one of the mechanisms through which CBD can achieve its anti-inflammatory effects.

### 2.1 eCB Signaling in ASD

Evidence is growing that many aberrant behaviors (in the sphere of emotionality, cognition and social disabilities) comprised in psychiatric NDDs, such as schizophrenia and ASD, can be improved by modifying the eCB signaling (Muguruza et al., 2013; Zamberletti et al., 2017). Indeed, there is a significant comorbidity between anxiety and ASD (van Steensel et al., 2011), and reduced serum levels of AEA, PEA and OEA has been found in children with ASD (Aran et al., 2019). The strong involvement of eCB signaling in the behavioral expression of deficits in social interaction has been elegantly demonstrated in the Src homology domain 3 and multiple ankyrin repeat domains 3 (SHANK3) mouse model of ASD-like behavior (Folkes et al., 2020). The model (i.e., *Shank3B* ^*–/*^) has been developed on the ground of the mutations and variants in SHANK3 encoding genes that have been found in patients with ASD (Vorstman et al., 2006; Boccuto et al., 2013; Kazdoba et al., 2016), which recapitulate loss of function of SHANK3 protein and ASD-like deficits in social behavior. The authors disclosed the role played by the basolateral amygdala-nucleus accumbens (BLA-NAc) circuit in the impairment of social behavior and elicitation of social avoidance, and demonstrated that an increase of social behavior was similarly achieved either by the inhibition of this BLA-NAc circuit in the *Shank3B* ^*–/*^ mouse, or by the increase of eCB signaling in both wild-type mice and ASD-like *Shank3B* ^*–/*^ mouse (Folkes et al., 2020). In particular, the increase in sociability was obtained by administering a selective MAGL inhibitor, thus demonstrating not only the involvement of eCB signaling in ASD pathogenesis but also the specific action produced by the increase of 2-AG mediated signaling. Even more recently, the same *Shank3* mutant mouse model has been used to evaluate the efficacy of a CBD-enriched cannabis oil in alleviating repetitive behavior and anxiety (Poleg et al., 2021). Along with a reduction of these ASD-like behaviors, including anxiety, this study evidenced the role of both CB_1_-mediated transmission and THC underlying the beneficial effect of medical cannabis-based treatment (Poleg et al., 2021). The anxiolytic activity of CBD has been well described in subjects with generalized social anxiety disorder (Crippa et al., 2011). Although not in lack of side effects, the conclusions of some studies completed by using CBD-enriched oil(s) in children with ASD (Fleury-Teixeira et al., 2019; Ponton et al., 2020; Loss et al., 2021) supports both the general improvement of symptoms (e.g., hyperactivity, anxiety, social abilities) and the idea of feasibility of CBD- and phytocannabinoid-based treatment for ASD. Similar conclusions have been outlined in an observational study reporting about 30% and 50% of significant and moderate symptoms improvement, respectively (Bar-Lev Schleider et al., 2019).

### 2.2 eCB Signaling, ASD and Gut Microbial Diversity: The Oxytocin Link

Considering the key involvement of eCB signaling in social bonding and sociality (Rapino et al., 2011) and the possibility to attenuate social phobia and social anxiety by increasing the eCB tone (Trezza et al., 2012; Micale et al., 2013), it was found that serum levels of AEA, PEA and OEA have been found decreased in subjects with ASD (Aran et al., 2019). In parallel, because of the involvement of the neuropeptide oxytocin in social cognition, social memories and facial recognition (Lopatina et al., 2018; Froemke and Young, 2021), there are studies in which oxytocin administration in patients with ASD has demonstrated either to improve some symptoms (e.g., emotion recognition, orientation towards human sounds) (Guastella et al., 2010; Lin et al., 2014) or to fail in eliciting significant differences between oxytocin-treated subjects and placebo group (Sikich et al., 2021). The eCB system functionally interact with the oxytocinergic transmission, which is known to exert anxiolytic effects and improve affiliation and social behavior (Yoon and Kim, 2020; Froemke and Young, 2021). Indeed, some central effects mediated by eCB congeners such as OEA have been demonstrated to depend on oxytocin signaling (Provensi et al., 2014), and several effects induced by the potentiation of oxytocin signaling such as reward-associated social interaction can be attenuated or suppressed by the antagonism at CB_1_ receptors (Wei et al., 2015). If AEA or OEA are directly involved in oxytocin-driven social reward (Wei et al., 2015), feeding (Provensi et al., 2014) and oxytocin secretion (Romano et al., 2013), there are then solid reasons to believe that, at least part of the pro-social effects obtained through oxytocin administration, could be attributed to eCB signaling. In this respect, it is worth quoting a recent functional magnetic resonance imaging study in which was observed a significant bilateral reduction of amygdala activity after subchronic intranasal oxytocin delivery, in positive correlation with symptoms relief, so that the lower the amygdala activity the greater the behavioral improvement (Bernaerts et al., 2020). Although the study by Folkes et al. (2020) did not discuss the results at the light of other connections, circuits, and functional interplay between different signaling systems, it is suggestive that the inhibition of the BLA-NAc circuit in the *Shank3B* ^*–/*^ ASD-like mouse model led to an improvement of social behavior and social avoidance. Moreover, the fact that sociability was boosted in this study *via* an increase of 2-AG signaling further strengthens the idea that the potential therapeutic provided by oxytocin delivery could be ascribed to the potentiation of eCB signaling.

The higher incidence of GI dysfunctions (e.g., abdominal pain, diarrhea but also constipation, food allergies and gluten-associated disorders) in children with autism than in control population is well documented (Ashwood et al., 2012; Xu et al., 2018), together with the interesting association between GI abnormalities, and severity of core symptoms such as more severe social withdrawal and anxiety in ASD (Hsiao, 2014). Moreover, as mentioned, microbiota dysfunction in terms of changes of gut bacterial composition, alteration of the integrity of the epithelial gut surface and abnormal intestinal permeability (“leaky gut”) are all recurrent clinical features in ASD population (Ristori et al., 2019). From this view, the CB_2_ receptor-mediated signaling may be an interesting player to connect eCB signaling, neuromodulatory activity of inflammatory processes, and ASD pathogenesis.

Thus, by focusing on the characterization of dysbiosis in autistic children is also possible to uncover the existence of correlations with altered oxytocin plasma levels in these patients and, indirectly, to reveal the existence of additional correlations linking the growth of pathogenic bacterial genera to the eCB system. By comparing gut microbiota of healthy controls and ASD subjects, a recent study has disclosed the existence of lower oxytocin plasma levels in ASD as well as an inverse correlation between oxytocin levels and increased abundance of genera bearing to the Clostridiales family (Huang et al., 2021). Among the bacterial genera present in higher abundance in the gut of ASD children, it was described an increase of *Erysipelotrichaceae* family, a butyrate-producing bacteria found abnormally grew in these patients (Huang et al., 2021). Interestingly, the only two bacterial families found reduced in gut microbiota of MAGL KO mice (Mgll^−/−^) were *Lactobacillaceae* and *Erysipelotrichaceae* (Dione et al., 2020), thus in animals showing increased levels of 2-AG and 2-AG congeners 2-monoacyl-glycerols (i.e., 2-MAGs) (Dione et al., 2020). In other terms, in biological systems where both 2-AG and 2-MAGs are elevated due to selective deletion of their hydrolyzing enzyme (i.e., MAGL) is possible to observe a decrease of some bacterial families that, by contrast, are elevated in ASD children with lower oxytocin plasma levels.

As mentioned, in addition to butyrate-producing bacteria, in subjects with ASD is frequently observed an increase of propionate-producing bacteria such as *clostridia* and *Desulfovibrio* bacteria (Finegold et al., 2010; De Angelis et al., 2013; de Angelis et al., 2015) that, when excessive, may induce neurotoxicity and neurotransmitter dysbiosis (Al-Salem et al., 2016). Accordingly, a diagnosis of autism is more frequent in subjects with propionic acidemia (Witters et al., 2016), and the experimental delivery (e.g., intracerebroventricular) of propionate has demonstrated to reproduce in rodents an entire set of ASD-like behaviors (Shultz et al., 2009; Meeking et al., 2020). Interestingly, CB_1_ receptor blockade can induce marked changes in the gut microbial community such as an increase of propionate production (Mehrpouya-Bahrami et al., 2017), and a downregulation of CB_1_ receptors has been disclosed in the post-mortem examination of brains of subjects with autism (Purcell et al., 2001). Hence, the overrepresentation in the gut community of ASD subjects of propionate-producing bacteria may contribute to the expression of ASD symptoms, and the downregulation of CB_1_ receptors contribute to propionate production and ASD pathophysiology.

### 2.3 ASD and the eCB Signaling-Gut Microbiota Reciprocal Influence

If ASD is a NDD accompanied by comorbid GI disorders and intestine inflammation, then we also know that eCBs and diet are reciprocally and metabolically linked and that the eCB system may be modulated not only by dietary factors and lifestyles (Bisogno and Maccarrone, 2014; Passani and Coccurello, 2016; Coccurello and Maccarrone, 2018), but also by gut microorganisms and selected bacterial taxa that can change circulating eCBs and regulate immune function and adaptive immunity (Castonguay-Paradis et al., 2020; Zheng et al., 2020). The eCB system is widely distributed all along the GI tract (Izzo et al., 2015) and the eCBs of the intestinal epithelium are functionally involved in the control of gut-brain signaling, thus influencing and being influenced by gut microbiota composition.

An elegant demonstration of this reciprocal influence is for instance showed *via* the association between anhedonia, as key diagnostic symptom in depression (Coccurello, 2019), and gut microbiota composition both in an animal model of depression-like behavior (Chevalier et al., 2020) and in a population cohort of volunteer twins (Minichino et al., 2021). The authors of this latter study used a model of analysis and a working hypothesis through which microbiota diversity can be exploited to predict changes in faecal and/or serum levels of eCBs and occurrence of anhedonia as trait of personality in the general population (Minichino et al., 2021). Notably, they found that PEA faecal (but not serum) levels mediates such association between microbial diversity and anhedonia incidence, further supporting the critical importance of the endocannabinoids-microbiota partnership for the understanding of symptoms that may anticipate the onset of a large set of neuropsychiatric and NDDs, including depression, substance abuse and ASD (Marrone and Coccurello, 2019). Paradigmatic is the case of *A. muciniphila*, a mucin-degrading Gram-negative bacterium able to regulate tight junctions and preserve gut health, whose activity opposes different pathogenetic mechanisms underlying metabolic diseases among which gut permeability, and for this also baptized “gut gatekeeper” (de Vos, 2017; Chelakkot et al., 2018). Indeed, *A. muciniphila* supplementation was shown to improve gut permeability, previously degraded by obesity-associated inflammation, and produce an increase of 2-AG intestinal levels (Everard et al., 2013). Later, the *A. muciniphila* beneficial effects have been attributed to a couple of eCB-derived lipids of the 2-AcGs family (i.e., 1-Palmitoyl-glycerol (1-PG) and 2-PG), and to their ability to bind the PPAR-α receptor (Depommier et al., 2021). Studies exploring changes of *A. muciniphila* density in the gut of ASD children have reported conflicting results. *A. muciniphila* abundance was found decreased according to the results by Wang et al. (2011), while was reported increased in another investigation (De Angelis et al., 2013). Nevertheless, here the main point is that important changes in the abundance of a bacterium that utilizes colonic mucin as substrate and protect from gut inflammation are frequently described in ASD, and that alterations of *A. muciniphila* can be mirrored by changes of eCB signaling.

Besides several studies reporting the beneficial effects of PEA supplementation on neurodegeneration (e.g., hippocampal damage) and social and non-social behaviors in different ASD-like rats and mice models (Bertolino et al., 2017; Cristiano et al., 2018; Herrera et al., 2018), an improvement of language skills following PEA supplementation has also been reported in two children with autism (Antonucci et al., 2015). Interestingly, PEA treatment in the ASD-like BTBR mouse model (Cristiano et al., 2018) also reduced pro-inflammatory cytokine production and intestinal permeability (“leaky” gut) at intestinal/colonic level. The PEA potential against clinical conditions involving GI inflammatory disorders has been shown in different models, as for experimental colitis in which the positive impact on inflammation and intestinal permeability has been attributed to the mediation of GPR55, PPAR-α and CB_2_ receptors (Borrelli et al., 2015). Among the interesting animal models of autistic-like behaviors (e.g., *fmr1* knockout) there is the exploitation of fragile X syndrome (FXS) and ASD comorbidity (Abbeduto et al., 2014) through the expansions mutations of *FMR1* gene, which greatly increase the incidence of ASD disabilities (Bailey et al., 2008). The seminal investigation of the eCB system in different animal models of FXS (Maccarrone et al., 2010b) has provided evidence that in mice lacking both the *FMR1*-encoding FXS protein FMRP (FMRP KO mice) and the regulatory brain cytoplasmic 1 RNA (BC1 KO mice) there is a severe alteration of the functional coupling between metabotropic glutamate 5 receptors (mGlu5Rs) and eCB signaling. Indeed, in double KO mice FMRP-BC1 was observed a marked disinhibition of mGlu5R-mediated activity leading to an increased activity of 2-AG-synthetizing enzyme diacylglycerol lipase (DAGL) and enhanced 2-AG levels (Maccarrone et al., 2010b). Of note, striatal levels of 2-AG were not found changed in FMRP KO mice but an increased activity of the enzyme responsible for 2-AG degradation (i.e., MAGL) was instead observed, thus suggesting a decrease of 2-AG tone/signaling in a brain region whose function is considered of key importance for autism pathophysiology (Fuccillo, 2016). Along the same line of thought, FMRP KO mice do not display the correct functional coupling between mGlu5R-dependent long-term depression and mGlu5-DAGL-dependent 2-AG release at the postsynaptic excitatory synapse, and inhibition of glutamate release upon 2-AG stimulation of presynaptic CB_1_ receptors (Jung et al., 2012)_._ Ultimately, this means that 2-AG production is downscaled, and blockade of 2-AG degradation through MAGL inhibition reinstated eCB-LTD in the striatum and normalized abnormal behavioral disinhibition in the elevated plus maze (Jung et al., 2012). More recently, a couple of studies showed that the increase of AEA signaling *via* pharmacological FAAH inhibition can counteract either social deficit (Wei et al., 2016) or aversive memory (Qin et al., 2015), not only in FXS animal model (i.e., *fmr1/*FMRP KO mouse), but also induce an improvement of social impairment in the BTBR mouse model of ASD-like behavior (Wei et al., 2016). To further corroborate the functional association between ASD and eCB signaling, and in line with the dysregulation of 2-AG metabolism found in different animal models of FXS (Maccarrone et al., 2010a; Jung et al., 2012), also the use of neurodevelopmental models of autism such as the prenatal exposure to valproic acid (VPA) has provided evidence for reduced DAGL-α activity in the cerebellum and concomitant increase of MAGL-dependent 2-AG degradation in the hippocampus (Kerr et al., 2013). The same study also shown that social exposure was able to induce an enhancement of hippocampal levels of NAEs such as PEA and OEA (Kerr et al., 2013). This study appears to match the framework emerging from the analysis of gut microbiota dysbiosis in depression-like behaviors in which has been described a reduced synthesis of 2-AG (Chevalier et al., 2020), as well as the aforementioned increase of 2-AG intestinal levels induced by *A. muciniphila* supplementation (Everard et al., 2013) and, although not always reported, the decrease of *A. muciniphila* abundance in children with autism (Wang et al., 2011). A brain decrease of NAPE-PLD expression and a parallel increase of FAAH-dependent AEA degradation following exposure to VPA prompts for the idea that defective AEA metabolism could, at least in part, account for the ASD-like deficits in social communication induced by VPA exposure (Servadio et al., 2016). Incidentally, the reduction of 2-AG (Kerr et al., 2013) and particularly of AEA (Servadio et al., 2016) brain levels described after exposure to VPA is reminiscent of the decrease of AEA serum levels observed in children with ASD (Aran et al., 2019), while pharmacological FAAH inhibition was shown to mitigate VPA-induced deficits in social behavior (Kerr et al., 2016).

Although with a significant variability of the bacterial taxa involved, gut microbial diversity is severely disrupted in ASD children. Overall, a reduced ratio of Bacteroidetes to Firmicutes in ASD patients is more frequently portrayed (De Angelis et al., 2013; Strati et al., 2017), as well as the fact that *Clostridium* and *Desulfovibrio* are generally found as pathologically dominant species (Finegold et al., 2010; de Angelis et al., 2015). By contrast, beneficial bacteria such as *Bifidobacterium* genera are commonly underrepresented in the altered bacterial profile of children with ASD (Adams et al., 2011; de Angelis et al., 2015).

Interestingly, changes in gut microbiota dysregulates the intestinal eCB system, and probiotic supplementation with *Lactobacillus acidophilus* induced an increase of CB_2_ receptor mRNA expression in mice colon (Rousseaux et al., 2007), which may be of importance for the above mentioned data concerning the upregulation of CB_2_ receptors in PBMCs of autistic patients (Siniscalco et al., 2013). The use of different *Lactobacillus* strains, in both experimental models of ASD-like behavior and clinical trials conducted on children with ASD, has provided increasing evidence that selected probiotics can ameliorate ASD symptoms. Here, it is worth remember the improvement of social deficits and the recovery of oxytocin levels in the paraventricular nucleus (PVN) of the hypothalamus obtained in offspring from obese mothers through the selective administration of *Lactobacillus reuteri* (Buffington et al., 2016). As noted, the alterations of the gut microbiota ecosystem in ASD patients include marked deficiencies in some bacterial species, such as *Bifidobacterium* species, which can be regulated by a class of probiotics/“psychobiotics” (Dinan et al., 2013) able to shape the diversity of commensal gut bacteria and affect several CNS functions and psychiatric diseases through different gut-to-brain routes of communication (e.g., enteric nervous system, vagal signaling, bacteria-derived metabolites and microbiota-neuroimmune axis) (Sarkar et al., 2016). The oral treatment with *Bacteroides fragilis* in offspring from mothers underwent immune activation improves both integrity of epithelial barrier and social-communicative deficits, restoring physiological gut composition and serum metabolites (Hsiao et al., 2013). Some of these *Bifidobacterium* species that are modulated by probiotic administration have been found strongly reduced in ASD pathophysiology, as demonstrated for the significant depletion of *Bifidobacterium longum (B. longum)* in young children with ASD (Coretti et al., 2018). Interestingly, the shift from Western diet to the consumption of Mediterranean diet was found to increase plasma OEA/PEA ratio, and such increase was observed to positively correlate with the growth of several microorganisms, included *B. longum* (Tagliamonte et al., 2021). Moreover, the administration of *B. longum* (under the form of probiotic mix) was shown to increase in zebrafish the intestinal mRNA expression of *Cnr1* and *Cnr2* genes thus providing molecular evidence of the contribution of eCB signaling, and in particular of the involvement of CB_2_ receptor-mediated signaling, in the anti-inflammatory effects achieved by probiotics administration (Gioacchini et al., 2017). Among other living bacteria and in addition to *B. longum*, the probiotic mix used included the *Lactobacillus acidophilus* and the *Bifidobacterium infantis*, and the study also showed a significant decrease of *faah* and *mgll* gene expression, thus suggesting a reduced degrading activity for both the enzymes and the potential increase of AEA- and 2-AG-mediated signaling (Gioacchini et al., 2017).

**FIGURE 3:**
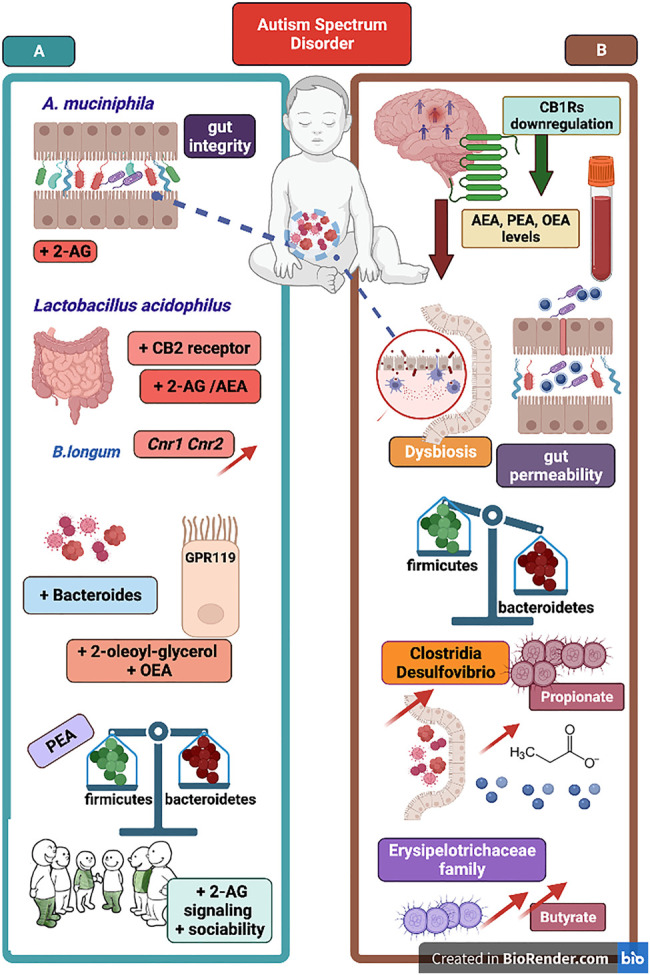
The figure portraits selected evidence in support of eCB signaling-gut microbiota relationship and representative mechanisms underlying the link between ASD pathophysiology and the eCB machinery. On the left panel **(A)**, *A. muciniphila* supplementation can improve gut permeability and recover gut integrity as well as increase 2-AG signaling. Supplementation of *Lactobacillus* acidophilus increase both 2-AG and AEA signaling and CB_2_ receptor mRNA expression in mice colon and in human epithelial cells. *B. Longum* supplementation is associated with increase of *Cnr1* and *Cnr2* genes intestinal expression. Below, an overall growth in Bacteroides population can produce an increase of eCB-like lipids (e.g., OEA and 2-OG) acting as GPR119 ligands. Recovery of gut microbial balance (e.g., Firmicutes to Bacteroides ratio) can be associated with PEA supplementation and an increase of 2-AG signaling cause an improvement of sociability. On the right panel **(B)**, downregulation of CB_1_ receptors found in brains of subjects with autism, and decrease of serum AEA, PEA and OEA levels in ASD patients. Below, some evidence of dysregulation of gut microbial community and reduced bacteria diversity. Gut dysbiosis in ASD is characterized by disrupted gut barrier integrity and increased gut permeability as well as by a general Firmicutes to Bacteroides imbalance. Next, the increase of Clostridia and Desulfovibrio bacteria as well as of Erysipelotrichaceae family are responsible of excessive propionate- and butyrate-producing bacteria, respectively. Boosting eCB tone and 2-AG and AEA signaling *via* prebiotics supplementation emerges as potential therapeutic *via* changes of gut microbial communities found defective in ASD patients.

*Bifidobacterium* species in general, and *B. longum* in particular, recruit multiple pathways and mechanisms to provide their well-known health benefit in humans (e.g., in a variety of GI disorders). Indeed, the impact of *B. longum* on intestinal ecology is triggered *via* different mechanisms among which the stimulation of the activity of butyrate-producing bacteria *via* acetate production (Sugahara et al., 2015). Butyrate (or butyric acid) is a short chain fatty acid (SCFA) that, likewise other SCFAs (e.g., acetate and propionate), is produced by the gut microbiota upon fermentation of non-digestible carbohydrates (Morrison and Preston, 2016). By exploiting its features of histone deacetylases (HDAC) inhibitor, butyrate treatment has been used in ASD-like mice models such as in the prenatal exposure to VPA (Takuma et al., 2014) and in the BTBR model of autism (Kratsman et al., 2016), in both cases describing improvements either in recognition memory or in social behavior. However, data incoming from ASD population are more controversial. On one hand, there are data collected in Chinese ASD patients in which are described lower than normal levels of faecal butyrate and reduced abundance of butyrate-producing bacteria (e.g., *Lachnospiraceae*) (Liu et al., 2019). On the other hand, there are data analysis in which a drastic reduction in *B. longum* microbiota colonization is reported together with an increase of the butyrate-producing bacterium *Faecalibacterium prausnitzii* (Coretti et al., 2018). Moreover, the severity of ASD symptoms appears to inversely correlate to *Faecalibacterium* genus (Ding et al., 2020), and a further confirmation of the increase of butyrate faecal level in children with ASD has been previously reported (Wang et al., 2012). Recently, it has been provided evidence that gut microbial metabolites, and specifically butyrate, can directly modulate the eCB tone at gut epithelial level (Hwang et al., 2021). Using different concentrations, the study showed that both eCBs synthetizing enzymes (i.e., NAPE-PLD and DAGL) were reduced by butyrate treatment in dose-dependent manner, and that in parallel butyrate was able to upregulate MAGL (but not FAAH) expression in the gut epithelial cells (Hwang et al., 2021). Accordingly, it seems conceivable that an excessive increase of butyrate level in children with ASD (Wang et al., 2012; Coretti et al., 2018) could contribute to reduce both AEA and 2-AG synthesis and, in particular, cause a depletion of 2-AG tone in the gut epithelium. Moreover, this point of view appears compatible with the decrease of AEA, PEA and OEA serum levels in children with ASD (Karhson et al., 2018; Aran et al., 2019). A recent investigation has compared the potential derangement of the eCB machinery in children with autism as well as in the ASD-like VPA animal model (Zou et al., 2021). Data from this study account for a general decrease of eCB signaling, which is corroborated by the increased expression of both mRNA and protein levels of CB_2_ receptors and eCB degrading enzymes FAAH and MAGL in PBMCs from autistic children (Zou et al., 2021). Of particular interest, the study not only confirmed the reduced blood concentration of AEA, PEA and OEA (Karhson et al., 2018; Aran et al., 2019) and the upregulation of CB_2_ receptors at PBMCs level (Siniscalco et al., 2013), but also reported a reduction of 2-AG serum levels in ASD patients (Zou et al., 2021). Here, Figure 3 can provide a snapshot of the reciprocal influence between eCB signaling and gut microbiota in ASD, as well as of the therapeutic potential that results from the boosting of eCB tone through the involvement of the gut microbial community.

Besides the effects of CBD in the *Shank3* mouse model of autism (Poleg et al., 2021), and the ASD-like neurophatological phenotype and deficits in social behavior described in models of prenatal exposure to VPA, in BTBR (Kratsman et al., 2016) and in FXS *fmr1* KO mice (Kazdoba et al., 2016), there is another link between mutations in Synapsin (SYN) genes and patients with comorbid ASD and epilepsy that is worth exploring (Fassio et al., 2011). Here, the relevance comes from the role that synapsins play in the regulation of synaptic transmission, plasticity, and neural development (Fornasiero et al., 2010). In particular, as demonstrated by the sophisticated assessment of ultrasonic vocalizations, synapsin II KO mice (i.e., *Syn*2) exhibited more marked deficits in social communication and therefore a substantial ASD-like phenotype (Michetti et al., 2017). Notably, from a functional point of view, synapsins also participate in eCB-induced neural plasticity so that the stimulation of CB_1_ receptors was shown to modulate the increase of synaptic vesicles by controlling synapsins phosphorylation and downstream activation of synaptic transmission (Patzke et al., 2021). Remarkably, restoration of gut dysbiosis by FMT was shown to normalize the reduced expression of presynaptic synapsin-1 expression in the APP/PS1 mouse model of Alzheimer’s disease (Wang et al., 2021), thus corroborating the use of ASD-like synapsin II KO mice for the study of the relationship between eCB signaling and gut microbiota in ASD development.

A further point worth of consideration, briefly outlined in the introduction, involves the deleterious effects of vitamin D deficiency on the reduction of microbial diversity were briefly mentioned as a convincing example of the eCB-microbiota reciprocal contamination, especially in the light of the beneficial results produced by PEA supplementation on the recovery of a balanced Firmicutes/Bacteroidetes ratio (Guida et al., 2020). Mice bearing a deletion of vitamin D receptor show dysbiotic microbial profile with a marked reduction of *A. muciniphila*, thus strengthens the importance of vitamin D signaling for the maintenance of intestinal integrity and eubiosys (Su et al., 2016). However, these examples could not be limited to the detrimental effects induced by vitamin D deficiency on dysbiosis but encompass the study of ASD pathogenesis. Indeed, accumulating evidence are in line with the notion that vitamin D deficiency during pregnancy can increase the risk of NDDs including ASD development (Lee et al., 2021), as well as that vitamin D supplementation might improve the expression of ASD symptoms (Principi and Esposito, 2020). Moreover, vitamin D deficiency during development has been associated to the risk of NPDs such as schizophrenia (McGrath et al., 2010). In support of a functional interplay between eCB signaling and vitamin D metabolism, there are the disruptive effects induced by high-dose systemic Δ9-THC administration in tasks simulating schizophrenia-like aberrant processing that were boosted by developmental vitamin D deficiency (Burne et al., 2014). A recent study addressing on astrocytes specific markers of neuronal aging and the anti-inflammatory potential of PEA (e.g., against reactive oxygen species and nitric oxide production) reported greater neuroprotective effects through the use of an innovative drug formulation consisting in PEA, alpha lipoic acid and vitamin D combination (Morsanuto et al., 2020). Not only the PEA/alpha lipoic acid/vitamin D formulation showed better cell viability, anti-inflammatory and anti-oxidant effects but also higher potency in the activation of CB_2_ receptors (Morsanuto et al., 2020), which have also been recently described to have a fundamental role in the preservation of social memory (Komorowska-Müller et al., 2021). Thus, to further support the importance of the endocannabinoids-microbiota reciprocal contamination for the understanding of ASD there are also the studies describing an association between vitamin D deficiency and risk of autism (Principi and Esposito, 2020) as well as the fact that PEA provided therapeutic beneficial in subjects with ASD (Khalaj et al., 2018) and the existence of a specific interaction between vitamin D metabolism, eCB signaling and changes in gut microbiota (Su et al., 2016; Guida et al., 2020; Morsanuto et al., 2020). Several key studies discussed in subchapters from Section 2.1 to Section 2.3 are summarized in Table 1.

**TABLE 1:** Summary table of the key studies showing involvement of eCB signaling in ASD.

eCB signaling in ASD	Subjects/System model	Major effects		Study
Circulating AEA, PEA and OEA	Children with ASD	Reduced serum levels unchanged ASD symptoms	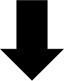	Aran et al. (2019)
MAGL inhibition and increase of eCB signaling in BLA-NAc circuit	SHANK3 mouse model	Decreased deficits in social behavior and social avoidance	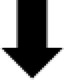	Folkes et al. (2020)
CBD-enriched oil	SHANK3 mouse model	Decreased ASD-like behavior (e.g., social anxiety)	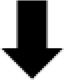	Poleg et al. (2021)
CBD-enriched oil	Children with ASD	Improvement of symptoms (hyperactivity, social abilities)	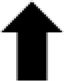	Ponton et al., (2020), Loss et al. (2021), Bar-Lev Schleider et al. (2019)
PEA supplementation	Children with ASD	Improvement language skills	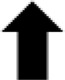	Antonucci et al. (2015)
Ultramicronized PEA + Luteolin coadministration	VPA-induced ASD-like mice	Improvement of social behavior	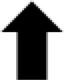	Bertolino et al. (2017)
Ultramicronized PEA + Luteolin coadministration	10 year old male children	Stereotypies decrease Improvement of ASD symptoms	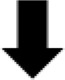	Bertolino et al. (2017)
FAAH inhibition/increase of AEA-signaling	*fmr1/*FMRP KO mouse model/ASD-like BTBR mouse model	Reversion of social deficit (both models)	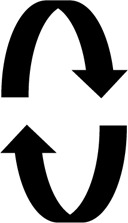	Wei et al. (2016)
Prenatal VPA exposure/effects in adolescent rats	ASD-like VPA rat model	Decreased DAGL-α activity/reduced 2-AG synthesis (cerebellum) Hippocampal enhancement of MAGL activity Social exposure induces AEA, PEA and OEA increase	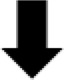 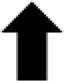 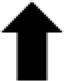	Kerr et al. (2013)
Prenatal VPA exposure	ASD-like VPA rat model	Decrease of NAPE-PLD expression/Increase of AEA degradation/Deficits social play and communication	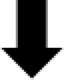 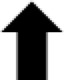 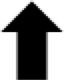	Servadio et al. (2016)
Pharmacological FAAH inhibition	ASD-like VPA rat model	Reduced VPA-induced deficits in social behavior	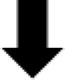	Kerr et al. (2016)
PBMCs	ASD patients	Up-regulation of CB_2_ receptors	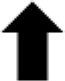	Siniscalco et al. (2013)

## 3 Endocannabinoids-Microbiota Reciprocal Contamination: ASD, Neuroinflammation and Immune Dysfunction

In spite of the quite elusive ASD etiology (Strathearn, 2009; Gyawali and Patra, 2019), there is a parallel field of investigation that started to look at some mechanisms of brain inflammation that may help to better understand this highly disabling NDD. In the search of pathogenetic mechanisms underlying ASD, it is not a mystery that the dysfunction of immune system is more than an ordinary hypothesis (Onore et al., 2012), which even include the possibility to consider ASD as an autoimmune disease (Hughes et al., 2018). If, as resident macrophages, microglia are the main innate immune cells of the brain (Hanisch and Kettenmann, 2007) and the eCB system is one of the major driver of the surveillance activity implemented by microglial cells (Mecha et al., 2015), then changes of eCB signaling can be responsible not only of healing and reparative processes but also of ASD pathogenesis. Aberrant changes in microglial cells morphology have been detected in postmortem brain of ASD subjects, and in particular a significant decrease in ramified microglia together with an increase in primed microglia in the gray matter of temporal cortex, thus supporting the idea that ASD is characterized by selective changes in microglial phenotypes (Lee et al., 2017). In a recent survey of the postmortem studies completed on the brain of subjects with ASD, the morphological alterations of microglia found in these samples were sufficient to account for the presence of neuroinflammation as etiological factor in ASD (Liao et al., 2020). CB_2_ receptors are mostly present on microglia and the relative level of expression of these receptors changes accordingly with the stage of neuropathology (Stella, 2009; Tanaka et al., 2020), and as function of the ability to regulate phagocytosis and transform the microglia phenotype from M1 to the anti-inflammatory M2 (Mecha et al., 2015). Remarkably, the eCB machinery and the switching between microglia phenotypes bidirectionally modulate each other. Indeed, in the M1 microglia phenotype both eCB receptors are downregulated while CB_2_ receptors are upregulated in both M2 activated subtypes M2a and M2c that, in turn, have been shown to promote the synthesis of 2-AG and AEA, respectively (Mecha et al., 2015). As evidenced by the actions of immunosuppressive drugs (Hughes et al., 2018), immune dysfunction is a key factor in ASD pathogenesis (Buffington et al., 2016) and CB_2_ receptors mediate many inflammatory processes and are highly expressed in immune cells (Chiurchiù et al., 2015; Turcotte et al., 2016). However, while in non-pathological conditions the expression of CB_2_ receptor is quite limited in the CNS, its expression is dramatically increased in microglial cells in case of neurodegenerative disorders such as Alzheimer’s disease (Benito et al., 2003; Benito et al., 2008), which is interpreted as a defense mechanism against neuroinflammation. CB_2_ receptors are particularly expressed in B lymphocytes (Lee et al., 2001) and, again, the upregulation of CB_2_ receptors might be interpreted as a defensive/protective mechanism to mediate immune response and suppression of inflammation. In parallel, the upregulation of CB_2_ receptors also demonstrate the tight link between alteration of CB_2_ signaling, immune dysfunction and ASD (Siniscalco et al., 2013). Remarkably, the enhancement of microglial phagocytic and migratory activity as well as the expression/activation of CB_2_ receptors are mechanisms involved in the anti-inflammatory effects and in the improvement of many ASD-like behaviors induced by the increase of PEA availability (Guida et al., 2017). Lower serum levels of not only PEA but also AEA and OEA have been detected in children with ASD (Aran et al., 2019), and both a reduction of mRNA levels of AEA-synthetizing enzyme NAPE-PLD, and an upregulation of CB_2_ receptors have been disclosed in peripheral blood mononuclear cells (PBMCs) from ASD children (Siniscalco et al., 2013). There is evidence that PEA supplementation can improve some specific ASD symptoms, especially when supplemented as nutritional adjuvant to pharmacological treatment. Thus, the concomitant administration of the atypical antipsychotic risperidone and PEA was shown to reduce ASD-associated aberrant behaviors such as hyperactivity/irritability in a greater extent with respect to risperidone therapy only (Khalaj et al., 2018).

Hence, ASD etiology has a recognized immune dysfunction, microglia are brain resident immune cells that are found morphologically altered in ASD, and eCB signaling and microglia polarization mutually interact and modulate each other. From this view, is of particular interest the ASD-like mouse model of maternal immune activation (MIA) that appears suitable to test the hypotheses of early developmental and inflammatory adverse events in ASD etiology (Bilbo et al., 2018), which are in some studies described in association with alterations of microglia motility during gestational period (Ozaki et al., 2020) and abnormal microglia density (Hadar et al., 2017). The focus on the immune dysregulation in ASD and the use of models such as MIA and prenatal exposure to VPA, allow us to emphasize the importance of both heritable and environmental factors during critical developmental periods. Interestingly, microglia isolated from animals that underwent the VPA model of ASD showed high reactive morphology and increased resistance to stimulation-induced phagocytosis (Traetta et al., 2021). The postnatal administration of the non-psychoactive phytocannabinoid cannabidivarin (CBDV) in rats underwent prenatal VPA exposure induced a recovery in the hippocampus of both glial fibrillary acidic protein (GFAP) and CD11b expression, as markers of astrocytes and microglia activation, respectively (Zamberletti et al., 2019). CBDV was also shown to relieve deficits in social behavior and hyperactivity and to upregulate hippocampal CB_2_ protein, thus supporting the possibility that CBDV-mediated boosting of the eCB machinery might not only reduce neuroinflammation but also contribute to the recovery of microglia morphological changes and facilitate the shift towards the anti-inflammatory M2 phenotype (Zamberletti et al., 2019). Several maternal infections (e.g., herpes simplex virus type 2, cytomegalovirus) (Mahic et al., 2017; Slawinski et al., 2018) or chronic inflammatory states (e.g., diabetes) (Xu et al., 2014) hitting the maternal environment increase the risk of ASD (Bilbo et al., 2018) and induce alterations of gut microbiota in the offspring, which may present an abnormal abundance of pro-inflammatory taxa (Ponzo et al., 2019).

The dramatic effects induced by altered gut microbial composition on microglia homeostasis are well documented by the consequences of the lack of maternal gut microbiota (e.g., GM mice) on microglia maturation, development, mature morphology and function (Fung et al., 2017; Thion et al., 2018). In particular, it has been provided evidence that maternal gut microbiota can directly sculpt intrauterine microglia dynamics and development, and increase the presence of ramified microglial cells, a morphology generally mirroring a major “resting state” (Thion et al., 2018). Notably, this study also establishes that maternal gut microbiota can drive microglia function in the offspring in sex-specific manner. Indeed, while male offspring showed an altered microglia morphology during the embryonic phase, in female offspring the alterations were more evident during adulthood (Thion et al., 2018), thus underscoring the impact of maternal gut ecosystem for early microglia development in males and contribute to explain the higher incidence of ASD in males and gender differences in ASD risk (Halladay et al., 2015). Here, we should consider the reduced ratio of Bacteroidetes to Firmicutes described in ASD subjects (De Angelis et al., 2013; Strati et al., 2017), and the further recently confirmed reduced level of *Bacteroides* in ASD gut microbiota (Cao et al., 2021). As recalled before, at the moment, *Bacteroides* are the only known microbiota bacterial species found to produce eCB-like lipids (i.e., *N*-acyl amides) similar to GPCR ligands, with a highest affinity for the GPR119 receptor whose best ligands are OEA and 2-OG (Cohen et al., 2017). The fact that MIA produced deficits in social behavior and that *Bacteroides fragilis* administration allowed recovering the impairment in social communication and integrity of intestinal permeability (Hsiao et al., 2013), may authorize to hypothesize that an increase of NAEs signaling and a shift of microglia phenotype may have a role in the recovery of ASD-like behaviors in models of immune dysregulation such as VPA and MIA. Moreover, if CBDV achieves its anti-inflammatory and prosocial behavioral effects after VPA exposure *via* the recovery of microglia morphological changes and through the upregulation of CB_2_ receptor in the hippocampus (Zamberletti et al., 2019), it should be noted that stimulation/upregulation of CB_2_ receptors is involved in the suppression of microglia activation (Ehrhart et al., 2005), in regulation of microglia recruitment and migration (Walter et al., 2003) as well as in the reduction of microglia-dependent release of pro-inflammatory factors and neurotoxicity (Komorowska-Müller and Schmöle, 2021). Hence, we might posit the existence of a vicious circle involving early immune dysfunction, microglia-driven neuroinflammation and neurotoxicity, alteration of gut microbiota and intestinal barrier integrity, disruption (generally reduced) of eCB- and CB_2_-mediated signaling and ASD pathogenesis. On the other side, to trigger an opposite virtuous circle both the administration of selected probiotics as well as non-psychoactive eCB compound and drugs capable to increase the activity of CB_2_ receptor may be considered together.

Alteration of maternal gut microbiota can be one pivotal mechanism responsible for MIA-induced alteration of sociability and neuroinflammation in the offspring (Kim et al., 2017). MIA- induced systemic inflammation and increase of interleukin-17a (IL-17a) plasma levels in pregnant mice has been in part ascribed to the activation of T helper 17 (T_H_17) cells, whose intestinal biogenesis is strongly stimulated by commensal organism known as segmented filamentous bacteria (SFB) (Farkas et al., 2015; Kim et al., 2017). Interestingly, mice lacking SFB did not develop MIA-induced ASD-like behavior and did show neither cortical alterations nor plasma increase in IL-17a levels. Thus, not only intestinal accumulation of SFB during pregnancy is detrimental for MIA-induced behavioral phenotype in the offspring, but the excessive differentiation of gut T_H_17 cells may increase the risk of a progeny with ASD. Abnormal T_H_17 activity and consequent IL-17 production are linked to multiple inflammatory, neurological and autoimmune diseases (Waite and Skokos, 2012; Milovanovic et al., 2020), and AEA, Δ9-THC and CBD treatment has shown efficacy against different T_H_17-driven diseases (Kozela et al., 2013; Jackson et al., 2014). In particular, eCBs are involved in the modulation of immune function and an increase of AEA signaling has been shown to suppress T_H_17-driven inflammation evoked by methylated Bovine Serum Albumin-induced delayed type hypersensitivity response *via* the upregulation of IL-10 secretion (Jackson et al., 2014). Other data in support of the involvement of eCBs in T_H_17-driven diseases have been provided for the suppressive effects obtained by THC and, above all, CBD administration upon IL-17 and IL-6 secretion, which has also been demonstrated to reduce the T_H_17-induced cell phenotype (Kozela et al., 2013). As observed, *Clostridium* genus is frequently found abundant in gut microbiota of subjects with ASD (Finegold et al., 2010; de Angelis et al., 2015) and *Clostridium-*derived metabolites [e.g., 3-(3-hydroxyphenyl)-3-hydroxypropionic acid] can be found elevated in urine samples from ASD patients (Shaw, 2010). Interestingly, based on gene sequence similarity, SFB are closely related to the *Clostridium* genus and their abnormal increase in the ileum of immunocompromised mice has been found counteracted by *Lactobacillus plantarum* administration (Fuentes et al., 2008). If SFB expression in the intestine epithelium stimulates T_H_17 cells development and contribute to the risk of ASD (Kim et al., 2017) but potentiation of eCB signaling is effective against T_H_17-driven diseases (Kozela et al., 2013; Jackson et al., 2014), then it is of further relevance that administration of *Lactobacillus plantarum* reduces SFB presence (Fuentes et al., 2008) and that *Lactobacillus plantarum* was included in the same probiotic mix observed to increase the expression of *Cnr1* and *Cnr2* genes in zebrafish intestinal epithelium (Gioacchini et al., 2017). Lastly, *Lactobacillus plantarum* administration was shown to normalize 2-AG hippocampal level in a mouse model of depression, and the restoration of the integrity of eCB signaling was accompanied by the renewal of hippocampal neurogenesis and alleviation of depressive-like behaviors (Chevalier et al., 2019).

**FIGURE 4:**
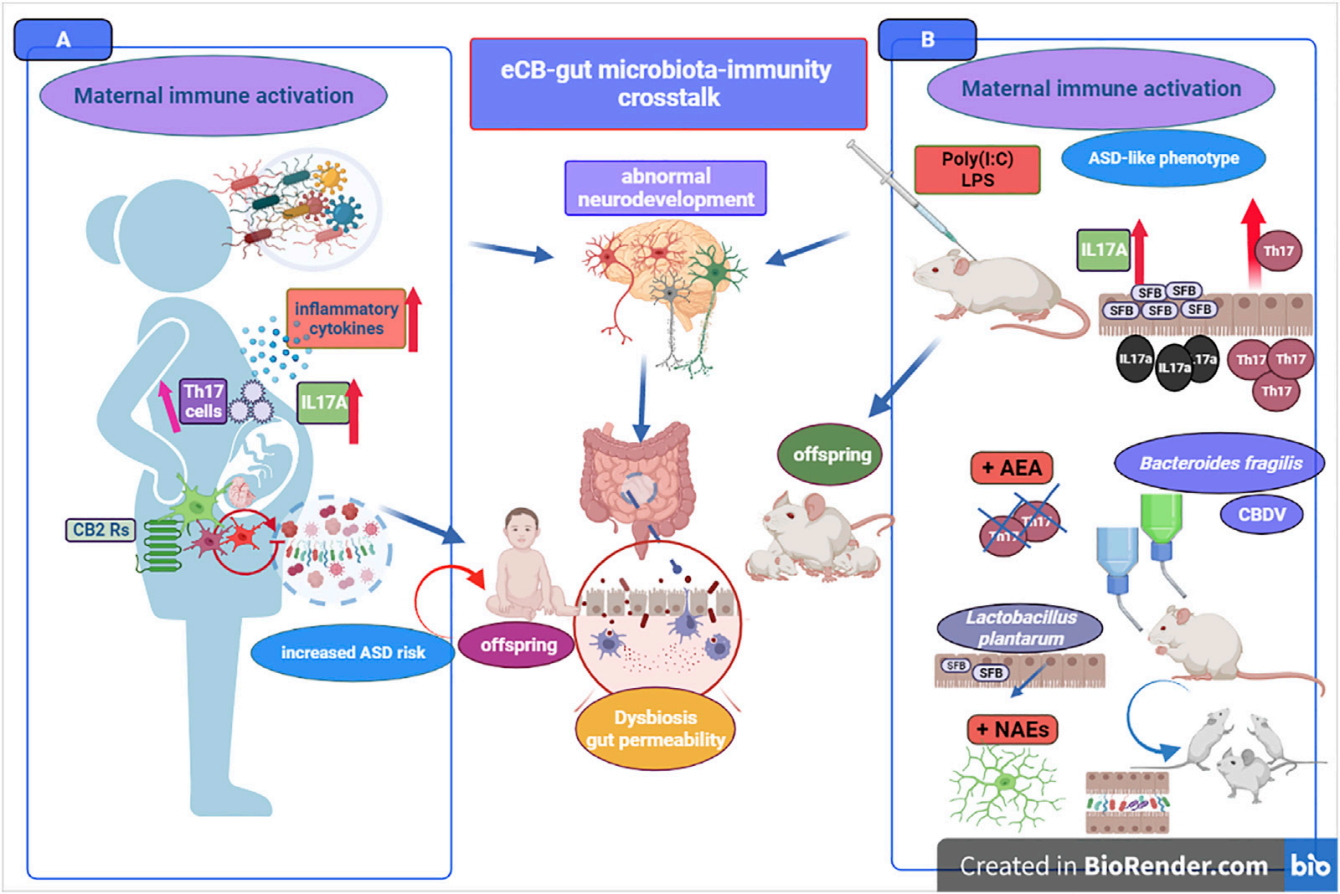
The figure portrays some key elements of the eCB signaling-gut microbiota-immune system crosstalk. Immune dysfunction can be a driving pathogenetic mechanism in ASD, with a strong deleterious impact on neuroinflammation, neurodevelopment and derangement of gut microbiota. Maternal immune activation (MIA) in pregnant mothers **(A)** and MIA-induced ASD-like mouse model **(B)** are both paradigmatic of increased ASD risk. Pathogens-induced deficits of systemic immune-regulation 1 provides an elevated level of explanation and isomorphism between ASD risk in humans and its simulation in mice. On the left panel **(A)**, exposure to several types of infectious agents (e.g., herpes simplex virus type 2, cytomegalovirus) during pregnancy is responsible of immune activation and immune-regulatory deficits (e.g., auto-antibody production and/or increase of Th17 cells) as well as of the overproduction of maternal inflammatory cytokines (e.g., IL17A). Immune-regulatory dysregulation during pregnancy contributes to maternal dysbiosis and altered microbiota composition, with detrimental impact on fetal microglia motility and states of activation of microglial cells. The neuroinflammatory maternal and fetal environment elevates the risk of abnormal neurodevelopment and ASD diagnosis with offspring showing marked derangement of gut microbiota. On the right panel **(B)**, the ASD-like MIA mouse model corroborates the hypothesis of early developmental and inflammatory adverse events in ASD etiology. Abnormal neurodevelopment, microglia morphological alterations and changes of offspring gut microbiota are common mechanisms in humans and mice models. Pro-inflammatory and immune challenges (e.g., Poly(I:C), LPS) trigger neurodevelopmental deficits in the offspring, with alteration of sociability and chronic neuroinflammation. For instance, microbiota segmented filamentous bacteria (SFB) produce an increase of Th17 activation (including IL17a production) but mice lacking SFB do not develop ASD-like symptoms. *L. plantarum* decreases SFB and supplementation with Bacteroides fragilis induces recovery from social communication deficit and damage to intestinal permeability. The crosstalk between eCBs bioactive lipids and ASD is further supported by the shift of microglia towards an anti-inflammatory phenotype and improve of ASD symptoms following the increase of NAEs-mediated signaling.

Thus, paradigmatic of the role of immune system not only as a link between gut microbiota and alteration of neurodevelopment in the offspring, but also of the possible overlapping mechanisms and reciprocal influence between eCB signaling and gut microbial ecology is the MIA construct. Some of these connections and few notions illustrated in this third chapter are exemplified in Figure 4. We mentioned before the dramatic changes of microglia maturation, density and/or morphology when gut microbiota underwent dysbiosis or reduced microbial diversity (Fung et al., 2017; Thion et al., 2018). However, despite the accumulating evidence in support of microbiota-microglia crosstalk, much less is known about the molecular messengers carrying to microglia the information concerning microbiota status. A potential answer to this issue has been provided by the demonstration that oral supplementation with a mix of SCFAs in GM mice produced a normalization of microglia in brain cortex (i.e., density stabilization) and a decrease of aberrant morphology and immaturity (Erny et al., 2015). Moreover, sodium butyrate treatment has been shown to put out the inflammation’s fire in brain-derived primary microglial cells after LPS challenge (Huuskonen et al., 2004), as well as more recently the administration of a SCFAs mixture was shown to decrease microglia-derived secretion of cytokines and cytotoxins relevant for Alzheimer’s disease pathogenesis (Wenzel et al., 2020). Hence, on this ground, SCFAs appear valid candidates as microbial metabolites carrying information from microbiota to microglial cells both for defense and upkeeping of microglia status. However, within the context of the present evaluation, it would also be particularly important to ascertain whether specific gut bacteria strains and/or microbial metabolites could affect eCB signaling. Well, the answer appears to be affirmative both for selected bacteria strains and SCFAs, and for butyrate in particular. As mentioned before, probiotics may trigger the intestinal expression of eCB receptors, and *Lactobacillus acidophilus* treatment was able to induce CNR2 mRNA expression on human epithelial cells. (Rousseaux et al., 2007). Indeed, oral administration in rats of *L. acidophilus* induced the expression of functional CB2 receptors, as corroborated by the fact that *L. acidophilus*-mediated analgesic effects were inhibited by CB_2_ receptors blockade (Rousseaux et al., 2007). SCFAs display anti-inflammatory activity, and a significant part of such activity is attributable to changes of eCB signaling. This is the main conclusion of a recent population study (Vijay et al., 2021) investigating the interaction between gut microbiota and eCB system in the secretion of well-characterized markers of inflammation. Interestingly, physical exercise was used to stimulate the growth of SCFA-producing bacteria, and by a formal analysis was possible to ascertain that physical exercise increased AEA, OEA and PEA, and that for instance AEA mediated about 30% of the effects of butyrate on TNF-α together with a positive association between 2-AG and OEA and anti-inflammatory cytokines such as IL-10. Additionally, the fact that both AEA and OEA were found positively correlated with SCFAs receptor expression (i.e., *FFAR2* receptor) is a critical evidence that the eCB system shapes microbiota composition and mediates part of the anti-inflammatory effects elicited by SCFA microbial mediators (Vijay et al., 2021). Several key studies discussed all along chapter 3 are summarized in Table 2.

**TABLE 2:** Summary table of the key studies involving eCB signaling and gut microbiota crosstalk in both patients with ASD and ASD-like animal models.

eCB signaling-microbiota partnership in ASD	Subjects/System model	Major effects		Study
Gut microbiota dysbiosis	Human HT-29 epithelial cells	Dysregulates the intestinal eCB system Increased intestinal cells CB2 receptor mRNA expression	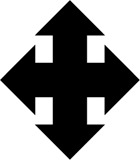 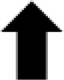	Rousseaux et al. (2007)
*Lactobacillus acidophilus* supplementation				
eCB and PEA faecal levels	General population	Prediction of the association between gut microbial diversity and anhedonia	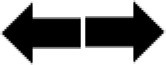	Minichino et al. (2021)
Prebiotic treatment: mucin-degrading Gram-negative bacterium	Children with ASD	*A. muciniphila* supplementation improves gut permeability/increases 2-AG intestine levels	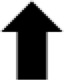	Everard et al. (2013)
Prebiotic treatment: mucin-degrading Gram-negative bacterium	Children with ASD	*A. muciniphila* supplementation provides beneficial effects dependent on eCB-derived lipids of the 2-AcGs family	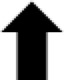	Depommier et al. (2021)
Mucin-degrading Gram-negative bacterium	Children with ASD	Decreased *A. muciniphila* abundance	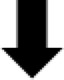	Wang et al. (2011)
Mucin-degrading Gram-negative bacterium	Children with ASD	Increased *A. muciniphila* abundance	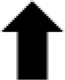	De Angelis et al. (2013)
Ultramicronized PEA + Luteolin coadministration	ASD-like BTBR mouse model	Decreased ASD-like repetitive behavior/pro-inflammatory cytokine production/ intestinal permeability/Increased sociability	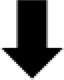	Cristiano et al.,(2018)
			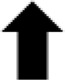	
*B. longum* probiotic mix (including *Lactobacillus acidophilus* and *B. infantis*) supplementation	Zebrafish	Increase intestinal mRNA expression of Cnr1 and Cnr2 genes Decrease of *faah* and mgll gene expression	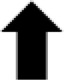 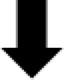	Gioacchini et al. (2017)
*B. fragilis* supplementation	ASD-like MIA model	Improves social-communicative deficits/integrity intestinal barrier	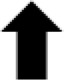	Hsiao et al. (2013)
*Bifidobacterium longum*	Children with ASD	ASD depletion of *B. longum* Decrease butyrate-producing bacteria	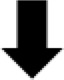	Coretti et al. (2018) Sugahara et al. (2015)
Butyrate treatment	ASD-like VPA and BTBR models	Improvement memory and social behavior	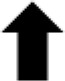	Takuma et al. (2014), Kratsman et al. (2016)
Butyrate and butyrate-producing bacteria	Children with ASD	Lower levels of butyrate and abundance of *Lachnospiraceae*	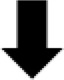	Liu et al. (2013)
Butyrate treatment (concentration-dependent effects)	Epithelial cell line Caco-2	Decrease eCBs synthetizing enzymes (i.e., NAPE-PLD; DAGL)	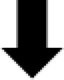	Hwang et al. (2021)
eCB system and signaling	Children with ASD vs ASD-like VPA murine model	eCB signaling FAAH and MAGL increased expression Decrease of 2-AG serum levels	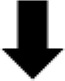 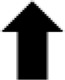 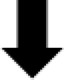	Zou et al. (2021)
Vitamin D	Vitamin D deficiency pregnancy Vitamin D supplementation	Risk of ASD Improve expression ASD symptoms	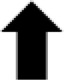 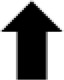	Lee et al. (2021) Principi and Esposito (2020)
PEA and vitamin D	Epithelial cell line Caco-2	CB2 receptor activation	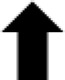	Morsanuto et al. (2020)
Microglial cells morphology	ASD subjects	Changes in microglial cells phenotype (e.g., decreased ramified microglia)	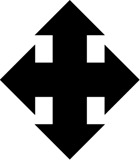	Lee et al. (2017)
PEA availability	Primary microglia cell culture	Increase microglial phagocytic/Migratory activity	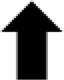	Guida et al. (2017)
CBDV supplementation	ASD-like VPA murine model	Microglia activation/Decrease deficit social behavior/Upregulation CB2 RS	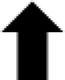 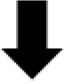 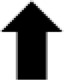	Zamberletti et al. (2019)
*Bacteroides*	ASD subjects	Reduced levels	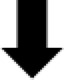	Cao et al. (2021)
*Bacteroides*	eCB-like production	High affinity GPR119 (2-OG and OEA)	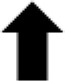	Cohen et al. (2017)
Systemic inflammation	ASD-like MIA murine mice Mice lacking SFB	Segmented filamentous bacteria (SFB) promotes TH17 intestinal biogenesis TH17-induced increase IL17-a plasma levels Failure of MIA-induced ASD-like symptoms	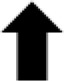 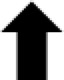 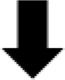	Farkas et al. (2015) Kim et al. (2017)
AEA, Δ9-THC, CBD administration	TH17-driven diseases	Microglia activation/Decrease deficits social behavior/Upregulation CB2 Rs	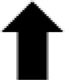 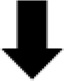 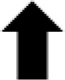	Kozela et al. (2019), Jackson et al. (2014)
*Lactobacillus plantarum* supplementation	Cecum and colon samples	Decrease SFB abundance	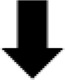	Fuentes et al. (2008)
SCFAs supplementation	Gut microbiota-eCB system interaction	Anti-inflammatory activity via eCB signaling Increase SCFA-dependent AEA, OEA and PEA levels AEA and OEA correlation with SCFAs receptor expression	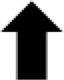	Vijay et al., (2021)
Physical exercise				

### 3.1 Summary and Conclusion

As for the understanding of neurodegenerative diseases (Armstrong, 2014), causes and effects are tightly intertwined in ASD, and no conclusive statements can be made on the primary or causal events underlying the eCBs-microbiota partnership in ASD etiology. Indeed, in spite of the considerable body of investigation, our current knowledge does not allow to determine whether the disruption of microbial ecosystem could be considered a primary event causing derangement of eCB signaling or, *vice-versa*, whether the unbalance of eCB system might trigger the disruption of gut microbial diversity descripted in ASD patients. The aspects so far examined might support both the lines of cause-effect relationship, thus either that a “major” gut derangement of microbial population produces downstream abnormal changes of eCB signaling and ASD-associated brain alterations (e.g., microglia-dependent neuroinflammation), or that dyshomeostasis of the eCB system contributes to alteration of gut microbial composition. Nevertheless, if the current updated appraisal of the endocannabinoids-gut microbiota partnership in ASD pathophysiology has provided sufficient evidence, this is a no way-out thinking.

Instead, adding the factor “eCB signaling” to the equation “ASD and gut microbiota” should help to disclose the role of eCB system as one of the major factors responsible of brain homeostasis. Loss of control exerted by the eCB system and ASD-associated abnormal variations in AEA, 2-AG and NAEs are factors that can help to fill the empty space between neurodevelopment, gut microbiota and brain inflammation. We reported extensive evidence (e.g., paragraphs in Sections 1.2, 2, 2.1, and 2.3) according to which ASD can be viewed as a NDD with a prominent state of brain inflammation and immune dysfunction. The detailed description of altered composition of intestinal microbiota in ASD-like models such as SHANK3, MIA, BTBR and VPA exposure provides several turning points where the study of the relationship between gut ecosystem and eCB signaling can be more elaborated.

Here, only a list of selected key evidence is reported, namely: 1) the recovery of social behavior in the *Shank3B* ^*–/*^ mouse is achieved by the increase of 2-AG mediated eCB signaling (*via* MAGL inhibition) or CBD-enriched cannabis oil (Folkes et al., 2020; Poleg et al., 2021); 2) the abnormal increase of selective bacteria family (e.g., *Erysipelotrichaceae*) in ASD patients with lower oxytocin levels and the parallel decrease of *Lactobacillaceae* and *Erysipelotrichaceae* families in animals with increased levels of 2-AG and 2-MAGs (Dione et al., 2020); 3) the possibility to predict eCBs changes such as PEA faecal levels through microbiota diversity (Minichino et al., 2021); 4) the association between gut permeability improvement, *A. muciniphila* supplementation, increase of 2-AG and 2-AcGs family intestinal levels (Everard et al., 2013; Depommier et al., 2021) and decreased *A. muciniphila* abundance in ASD children (Wang et al., 2011); 5) the beneficial effects of PEA supplementation on neuroinflammation and social behaviors in ASD-like BTBR mouse model (Cristiano et al., 2018); 6) the possibility to counteract social deficit by increasing AEA signaling both in FXS (i.e., *fmr1/*FMRP KO mouse) and BTBR models of ASD-like behavior (Wei et al., 2016); 7) the evidence for reduced DAGL-α activity and increase of 2-AG degradation in the neurodevelopmental VPA model (Kerr et al., 2013); 8) the increase AEA degradation following exposure to VPA (Servadio et al., 2016) and the mitigation of social communication deficits after FAAH inhibition in the same model (Kerr et al., 2016); 9) the possibility to increase colon expression of CB_2_ receptor mRNA after *L. acidophilus* supplementation (Rousseaux et al., 2007) mirroring the upregulation of CB_2_ receptors in PBMCs of ASD subjects (Siniscalco et al., 2013); 10) the possibility to increase the intestinal mRNA expression of *Cnr1* and *Cnr2* genes as well as decrease of *faah* and *mgll* gene expression after *B. longum*, *L. acidophilus* and *Bifidobacterium infantis* supplementation (Gioacchini et al., 2017); 11) the existence of a functional interaction between eCB signaling, vitamin D metabolism, and changes in gut microbiota in ASD (Su et al., 2016; Guida et al., 2020; Morsanuto et al., 2020) 12) the ability of selected probiotics such as *B. infantis* to improve KP metabolism (Desbonnet et al., 2008), and also stimulate the expression of *Cnr1* and *Cnr2* genes in zebrafish intestinal epithelium (Gioacchini et al., 2017) and improve autistic core symptoms (Grossi et al., 2016); 13) the microglia alterations in density and morphology observed in ASD patients and ASD-like MIA mouse model (Hadar et al., 2017; Bilbo et al., 2018), and the possibility to recover social behavior, microglia activation and M2 phenotype by enhancing the eCB machinery; 14) the fact that intestinal SFB expression stimulates T_H_17 cells and increase ASD risk (Kim et al., 2017) but enhancement of eCB signaling improves T_H_17-driven diseases (Kozela et al., 2013; Jackson et al., 2014) and *Lactobacillus plantarum* administration decreases SFB presence and contribute to the expression of *Cnr1* and *Cnr2* genes (Gioacchini et al., 2017); 15) the fact that SCFA-producing bacteria are stimulated by physical exercise and that the parallel increase of AEA, OEA and PEA mediate the butyrate-dependent effects on anti-inflammatory activity (Vijay et al., 2021); 16) lastly, the fact that selected gut microbiota bacteria (e.g., *Bacteroides*) have been found capable to produce eCB-like molecules such as commendamide (N-acyl-3-hydroxypalmitoyl-glycine), which is an agonist at GPR132 (Cohen et al., 2015), and N-oleoylserinol that is an agonist of the eCB receptor GPR119 (Cohen et al., 2017).

Finally, a still unexplored area of investigation might emerge from the more extensive understanding of the role of astrocytes in ASD pathogenesis (Gzielo and Nikiforuk, 2021), and in particular by focusing the study on astroglial CB_1_ receptors and lactate signaling in astrocyte-neuron communication (i.e., astrocyte-neuron lactate shuttle, ANLS) as well as on the perturbation of lactate levels and the alterations of lactate-producing bacteria in gut microbiota of subjects with ASD. There is indeed evidence that these aspects could be re-examined at the light of their functional relationship with the ASD pathophysiology. Lactate is known to act not only as signaling molecule between astrocytes and neurons where is required to meet energy needs and maintain brain homeostasis (i.e., metabolic coupling and ANLS) (Pellerin and Magistretti, 1994; Mason, 2017), but also as signaling molecule involved in memory formation (Suzuki et al., 2011) and in schizophrenia-like aberrant behavior (Sun et al., 2021). Several clinical surveys report higher blood lactate levels and higher lactate-to-pyruvate ratio in ASD (Oh et al., 2020), which are biochemical changes that could at least in part attributed to the dysregulation of the WNT/β-catenin pathway (Vallée and Vallée, 2018) and/or to reduction of gut bacteria responsible for lactate fermentation such as the decrease of *Veillonellaceae* described in subjects with ASD (Kang et al., 2013). Moreover, in some cases, the higher presence of lactate-producing bacteria might contribute to the reduction of SCFAs levels, and in particular of butyrate, whose consequences (e.g., inflammation susceptibility) have been emphasized earlier. Although with important exceptions (Coretti et al., 2018), in ASD has been reported not only a reduction of SCFA- and butyrate-producing bacteria, but in particular a reduction of the genera *Anaerostipes* and *Eubacterium*, (Liu et al., 2019; Cao et al., 2021), which are both essential for lactate-to-SCFAs conversion and prevention of lactate accumulation (Duncan et al., 2004).

In conclusion, the idea that gut microbiota can determine and underlie ASD pathophysiology is increasingly accepted, and in this critical review we have provided extensive evidence for gut dysbiosis, reduced microbial diversity, increased inflammation of gut epithelium, selective derangement of microbial population and gut ecosystem in ASD patients, aberrant immune profile, role of SCFAs and microbiota-derived metabolites and effects of probiotic intervention. In parallel, we discussed the literature focused on the emerging issue of the involvement of the eCB machinery in ASD and ASD-associated alterations of social behavior, as well as on the demonstration in ASD patients and in ASD-like animal models of a general decline in eCB signaling, and the potential therapeutic applications thereof in terms of both achievements and failures. A great attention has been addressed to the perspective to consider together the two aspects, and the possibility that the eCBs-microbiota partnership might offer novel pathways of investigation and synergistic options for therapeutic intervention, both pharmacological and nutritional, especially with the aim to improve the quality of life of patients and their caregivers.
