# Supplementary material for: The Endocannabinoids-Microbiota Partnership in Gut-Brain Axis Homeostasis: Implications for Autism Spectrum Disorders
Source: Front Pharmacol. 2022 Jun 3;13:869606. doi: 10.3389/fphar.2022.869606 (PMC9204215; doi:10.3389/fphar.2022.869606)
Supplement: Supplementary file 1 [file DataSheet2.PDF]

eCB signaling-microbiota partnership in ASD	bjects /System model	Major Effects		Study
Gut microbiota dysbiosis<br><br>*Lactobacillus acidophilus* supplementation	Human HT-29 epithelial cells	Dysregulates the intestinal eCB system<br><br>Increased intestinal cells CB2 receptor mRNA expression	↕↔<br><br>↑	**Rousseaux et al., 2007**
eCB and PEA faecal levels	General population	Prediction of the association between gut microbial diversity and anhedonia	↔	**Minichino et al., 2021**
Prebiotic treatment: mucin-degrading Gram-negative bacterium	Children with ASD	A. muciniphila supplementation improves gut permeability/ increases 2-AG intestine levels	↑	**Everard et al., 2013**
Prebiotic treatment: mucin-degrading Gram-negative bacterium	Children with ASD	*A. muciniphila* supplementation provides beneficial effects dependent on eCB-derived lipids of the 2-AcGs family	↑	**Depommier et al., 2021**
Mucin-degrading Gram-negative bacterium	Children with ASD	Decreased *A. muciniphila* abundance	↓	**Wang et al., 2011**
Mucin-degrading Gram-negative bacterium	Children with ASD	Increased A. muciniphila abundance	↑	**De Angelis et al., 2013**

Ultramicronized PEA + Luteolin coadministration	ASD-like BTBR mouse model	Decreased ASD-like repetitive behavior/ pro-inflammatory cytokine production/ intestinal permeability/<br><br>Increased sociability	↓<br><br>↑	**Cristiano et al., 2018**
B. longum probiotic mix (including *Lactobacillus acidophilus and B. infantis*) supplementation	*zebrafish*	Increase intestinal mRNA expression of Cnr1 and Cnr2 genes<br><br>Decrease of *faah* and mgll gene expression	↑<br><br>↓	**Gioacchini et al., 2017**
B. fragilis supplementation	ASD-like MIA model	Improves social-communicative deficits/ integrity intestinal barrier	↑	**Hsiao et al., 2013**
Bifidobacterium longum	Children with ASD	ASD depletion of *B. longum*<br><br>Decrease butyrate-producing bacteria	↓	**Coretti et al., 2018**<br><br>**Sugahara et al., 2015**
Butyrate treatment	ASD-like VPA and BTBR models	Improvement memory and social behavior	↑	**Takuma et al., 2014**<br><br>**Kratsman et al., 2016**
Butyrate and butyrate-producing bacteria	Children with ASD	Lower levels of butyrate and abundance of *Lachnospiraceae*	↓	**Liu et al., 2013**

Butyrate treatment<br><br>(concentration-dependent effects)	Epithelial cell line Caco-2	Decrease eCBs synthetizing enzymes (i.e., NAPE-PLD; DAGL)	↓	**Hwang et al., 2021**
eCB system and signaling	Children with ASD vs ASD-like VPA murine model	eCB signaling<br><br>FAAH and MAGL increased expression<br><br>Decrease of 2-AG serum levels	↓<br><br>↑<br><br>↓	**Zou et al., 2021**
Vitamin D	Vitamin D deficiency pregnancy<br><br>Vitamin D supplementati on	Risk of ASD<br><br>Improve expression ASD symptoms	↑<br><br>↑	**Lee et al., 2021**<br><br>**Principi and Esposito, 2020**
PEA and vitamin D	Epithelial cell line Caco-2	CB2 receptor activation	↑	**Morsanuto et al., 2020**
Microglial cells morphology	ASD subjects	Changes in microglial cells phenotype (e.g., decreased ramified microglia)	✥	**Lee et al., 2017**
PEA availability	Primary microglia cell culture	Increase microglial phagocytic/<br><br>Migratory activity	↑	**Guida et al., 2017**
CBDV supplementation	ASD-like VPA murine model	Microglia activation/<br><br>Decrease deficit social behavior/<br><br>Upregulation CB2 RS	↑<br><br>↓<br><br>↑	**Zamberletti et al., 2019**
Bacteroides	ASD subjects	Reduced levels	↓	**Cao et al., 2021**

Bacteroides	eCB-like production	High affinity GPR119<br><br>(2-OG and OEA)	↑	**Cohen et al., 2017**
Systemic inflammation	ASD-like MIA murine mice<br><br>Mice lacking SFB	Segmented filamentous bacteria (SFB) promotes TH17 intestinal biogenesis<br><br>TH17-induced increase IL17-a plasma levels<br><br>Failure of MIA-induced ASD-like symptoms	↑<br><br>↑<br><br>↓	**Farkas et al. 2015**<br><br>**Kim et al., 2017**
AEA, Δ9-THC, CBD administration	TH17-driven diseases	Microglia activation/<br><br>Decrease deficits social behavior/<br><br>Upregulation CB2 Rs	↑<br><br>↓<br><br>↑	**Kozela et al., 2019;**<br><br>**Jackson et al., 2014**
Lactobacillus plantarum supplementation	Cecum and colon samples	Decrease SFB abundance	↓	**Fuentes et al., 2008**
SCFAs supplementation<br><br>Physical exercise	Gut microbiota-eCB system interaction	Anti-inflammatory activity via eCB signaling<br><br>Increase SCFA-dependent AEA, OEA and PEA levels<br><br>AEA and OEA correlation with SCFAs receptor expression	↑	**Vijay et al., 2021**

TABLE 2
Summary table of the key studies involving eCB signaling and gut microbiota crosstalk in both patients with ASD and ASD-like animal models.
