# Supplementary material for: The Endocannabinoids-Microbiota Partnership in Gut-Brain Axis Homeostasis: Implications for Autism Spectrum Disorders
Source: Front Pharmacol. 2022 Jun 3;13:869606. doi: 10.3389/fphar.2022.869606 (PMC9204215; doi:10.3389/fphar.2022.869606)
Supplement: Supplementary file 2 [file DataSheet3.PDF]

2-arachidonoylglycerol (2-AG)
2-acylglycerols (2-AcGs),
2-AcGs family (1-Palmitoyl-glycerol (1-PG) and 2-PG),
2-oleoyl-glycerol (2-OG),
2-linoleoyl-glycerol (2-LG)
2-monoacyl-glycerols (2-MAGs)
2-palmitoylglycerol (2-PG)
α-linolenic acid (ALA)
anandamide (AEA)
Akkermansia muciniphila (*A. muciniphila*)
arachidonic acid (ARA), autism spectrum disorders (ASD)
avoidant/restrictive food disorder (ARFID)
Δ-9-tetrahydrocannabinol (THC)
cannabidiol (CBD)
cannabidivarin (CBDV),
diacylglycerol lipase (DAGL)
docosahexaenoic acid (DHA)
eicosapentaenoic acid (EPA)
eating disorders (EDs)
endocannabinoids (eCBs)
fatty acid amide hydrolase (FAAH)
fecal microbiota transplantation (FMT)
fragile X syndrome (FXS)
G protein-coupled receptors (GPCRs)
G protein-coupled receptor 55 (GPR55)
germ-free (GF)
glial fibrillary acidic protein (GFAP)
glucagon-like peptide-1 (GLP-1)
interleukin-17a (IL-17a)
linoleic acid (LA)
liver-expressed antimicrobial peptide 2 (LEAP2)
maternal immune activation (MIA)
monoacylglycerol lipase (MAGL),
N-acyl-ethanolamines (NAEs)
N-acyl-phosphatidylethanolamine-specific phospholipase D (NAPE-PLD)
N-arachidonoylserotonin (AA-5-HT)
N-docosahexaenoyl-ethanolamine (DHEA)
N-eicosapentaenoyl-ethanolamine (EPEA)
N-oleoylethanolamine (OEA),
N-palmitoylethanolamine (PEA)
N-linoleoylethanolamine (LEA)
N-stearoylethanolamide (SEA)
neurodevelopmental disorders (NDDs)
neuropsychiatric disorders (NPDs)
oleic acid (OA)
palmitic acid (PA)

peroxisome proliferator-activated receptor-α (PPAR-α)
peroxisome proliferator-activated receptor- γ (PPAR- γ)
peroxisome proliferator-activated receptor- δ (PPAR-δ)
polyunsaturated *n*-6 fatty acids (*n*-6 PUFAs)
transient receptor potential vanilloid type-1 (TRPV1)
segmented filamentous bacteria (SFB)
short chain fatty acid (SCFA)
Src homology domain 3 and multiple ankyrin repeat domains 3 (SHANK3)
type 1 cannabinoids receptors (CB_1)
type 2 cannabinoids receptors (CB_2)
valproic acid (VPA)
Western diet (WD)
