# Supplementary material for: The Endocannabinoids-Microbiota Partnership in Gut-Brain Axis Homeostasis: Implications for Autism Spectrum Disorders
Source: Front Pharmacol. 2022 Jun 3;13:869606. doi: 10.3389/fphar.2022.869606 (PMC9204215; doi:10.3389/fphar.2022.869606)
Supplement: Supplementary file 3 [file DataSheet1.PDF]

eCB signaling in ASD	Subjects /System model	Major Effects		Study
Circulating AEA, PEA and OEA	Children with ASD	Reduced serum levels unchanged ASD symptoms	↓	**Aran et al., 2019**
MAGL inhibition and increase of eCB signaling in BLA-NAc circuit	SHANK3 mouse model	Decreased deficits in social behavior and social avoidance	↓	**Folkes et al., 2020**
CBD-enriched oil	SHANK3 mouse model	Decreased ASD-like behavior (e.g., social anxiety)	↓	**Poleg et al., 2021**
CBD-enriched oil	Children with ASD	Improvement of symptoms (hyperactivity, social abilities)	↑	**Ponton et al., 2020**<br>**Loss et al., 2021**<br>**Bar-Lev Schleider et al., 2019**
PEA supplementation	Children with ASD	Improvement language skills	↑	**Antonucci et al., 2015**
Ultramicronized PEA + Luteolin coadministration	VPA-induced ASD-like mice	Improvement of social behavior	↑	**Bertolino et al., 2017**
Ultramicronized PEA + Luteolin coadministration	10 year old male children	Stereotypies decrease Improvement of ASD symptoms	↓	**Bertolino et al., 2017**
FAAH inhibition/increase of AEA-signaling	*fmr1*/FMRP KO mouse model/ ASD-like BTBR mouse model	Reversion of social deficit (both models)	↻	**Wei et al., 2016**

Prenatal VPA exposure/effects in adolescent rats	ASD-like VPA rat model	Decreased DAGL-α activity/reduced 2-AG synthesis (cerebellum)<br><br>Hippocampal enhancement of MAGL activity<br><br>Social exposure induces AEA, PEA and OEA increase	↓<br><br>↑<br><br>↑	**Kerr et al., 2013**
Prenatal VPA exposure	ASD-like VPA rat model	Decrease of NAPE-PLD expression/<br><br>Increase of AEA degradation/<br><br>Deficits social play and communication	↓<br><br>↑<br><br>↑	**Servadio et al., 2016**
Pharmacological FAAH inhibition	ASD-like VPA rat model	Reduced VPA-induced deficits in social behavior	↓	**Kerr et al., 2016**
PBMCs	ASD patients	Up-regulation of CB_2 receptors	↑	**Siniscalco et al., 2013**

TABLE 1
Summary table of the key studies showing involvement of eCB signaling in ASD.
